# Differential and Common Signatures of miRNA Expression and Methylation in Childhood Central Nervous System Malignancies: An Experimental and Computational Approach

**DOI:** 10.3390/cancers13215491

**Published:** 2021-10-31

**Authors:** George I. Lambrou, Myrto Poulou, Krinio Giannikou, Marios Themistocleous, Apostolos Zaravinos, Maria Braoudaki

**Affiliations:** 1 Choremeio Research Laboratory, First Department of Pediatrics, National and Kapodistrian University of Athens, 11527 Athens, Greece; glamprou@med.uoa.gr; 2 Department of Medical Genetics, Medical School, National and Kapodistrian University of Athens, 15772 Athens, Greece; mrpoulou@med.uoa.gr; 3 Cancer Genetics Laboratory, Division of Pulmonary and Critical Care Medicine and of Genetics, Brigham and Women’s Hospital and Harvard Medical School, Boston, MA 02115, USA; krinio.giannikou@gmail.com; 4 Department of Neurosurgery, “Aghia Sofia” Children’s Hospital, 11527 Athens, Greece; mthemistocleous@gmail.com; 5 Department of Life Sciences, School of Sciences, European University Cyprus, Nicosia 2404, Cyprus; 6 Basic and Translational Cancer Research Center (BTCRC), Cancer Genetics, Genomics and Systems Biology Group, European University Cyprus, Nicosia 1516, Cyprus; 7 Department of Life and Environmental Sciences, School of Life and Health Sciences, University of Hertfordshire, Hertfordshire AL10 9AB, UK

**Keywords:** methylation, childhood CNS tumors, miRNA, mRNA, microarray

## Abstract

**Simple Summary:**

Epigenetic mechanisms, that are modifications of the genome without the presence of mutations, are known to play a crucial role in central nervous system (CNS) tumors during childhood. Two well-known epigenetic regulatory mechanisms include methylation and miRNA regulatory mechanisms. Therefore, in the present study we have investigated the presence of methylated genes in childhood CNS tumors, along with miRNA expression. We have searched for correlations between gene methylation and miRNA expression. In addition, we have investigated mRNA expression in order to search for possible miRNA targets. Such approaches could prove useful for the improvement of CNS tumor prognosis, as well as for the discovery of new therapeutic targets.

**Abstract:**

Epigenetic modifications are considered of utmost significance for tumor ontogenesis and progression. Especially, it has been found that miRNA expression, as well as DNA methylation plays a significant role in central nervous system tumors during childhood. A total of 49 resected brain tumors from children were used for further analysis. DNA methylation was identified with methylation-specific MLPA and, in particular, for the tumor suppressor genes CASP8, RASSF1, MGMT, MSH6, GATA5, ATM1, TP53, and CADM1. miRNAs were identified with microarray screening, as well as selected samples, were tested for their mRNA expression levels. CASP8, RASSF1 were the most frequently methylated genes in all tumor samples. Simultaneous methylation of genes manifested significant results with respect to tumor staging, tumor type, and the differentiation of tumor and control samples. There was no significant dependence observed with the methylation of one gene promoter, rather with the simultaneous presence of all detected methylated genes’ promoters. miRNA expression was found to be correlated to gene methylation. Epigenetic regulation appears to be of major importance in tumor progression and pathophysiology, making it an imperative field of study.

## 1. Introduction

Cancer is one of the leading fatal diseases in the western world. Tumors of the central nervous system (CNS), are considered to be a complex, heterogeneous disease that is often fatal. Scientific knowledge gained through intense research, have not yet been translated into similar improvements in tumor patients. CNS tumors still remain a critical health condition, challenging both the patient as well as the health professionals. CNS tumors are a pathological condition of unknown etiology that lead to the formation of solid mass growing uncontrollably. They are characterized by abnormalities and disorders in the proliferation, differentiation, and gene expression of a particular cell population, which usually results in abnormal cell function. Although the etiology of CNS tumors is not fully understood, there are specific factors that increase the risk of developing the disease. Some of them include the exposure to radiation, genomic factors, exposure to mutagenic chemicals, and infection by specific viruses.

Tumors in the central nervous system (CNS) during childhood, are the second most frequent tumor types and are considered to be the most fatal of all neoplasms. CNS tumors, are almost unique due to their anatomical position and the perils that such a location includes. In particular, examples of CNS tumors include pilocytic astrocytomas (PA), ependymomas (EP), medulloblastoma (MB), atypical teratoid/rhabdoid tumors (ATRT), dysembryoplastic neuroepithelial tumors (DNETs), and others. The aforementioned tumors are most frequent in children, where their clinical presentation not only concerns the nervous tissue but can also “infiltrate” the cerebrospinal fluid (CSF) and the subarachnoid space. The classification of CNS tumors has been extensively studied and the World Health Organization (WHO) has produced an official report on the subject. The classification of CNS tumors with respect to the “WHO Classification of Tumors of the Central Nerbous System” has been described previously [1,2].

### 1.1. CNS Tumor Biomarkers

An important diagnostic tool for the determination and staging of CNS tumors is still the investigation of protein biomarkers, which are detected by immunohistochemistry (IHC) [3,4,5]. Numerous protein markers are used for the determination of tumor staging, where some include glial fibrillary acidic protein (GFAP), P38, neuromicrofilaments (NF), tumor protein p53 (p53), Bcl2 apoptosis regulator (BCL2), β-tubulin III (TUBB3), RNA binding Fox-1 homolog 3 (Neu-N), β-catenin (CTNNB1), pre-mRNA-splicing factor ini1 (INI1), marker of proliferation Ki-67 (MKI67), keratin, mucin 1 cell surface associated (MUC1 or EMA), epidermal growth factor receptor (EGFR), P27, S100 calcium binding protein (S100), actin, desmin (DES), myelin (MBP), and others. Immunohistochemistry consists of an invaluable tool for the differential diagnostic process and the accurate determination of tumors of the CNS [6]. Out of the numerous immunohistochemical biomarkers available, several have been highlighted as “independent prognostic markers” [7,8]. For example, the ki-67 is an antigen whose expression is related to the tumors’ aggressiveness in the malignancies of the CNS [7,8]. The biological properties of ki-67 made it a significant biomarker in terms of tumor prognosis. Currently, the most prevalent therapeutic approach concerns the craniospinal irradiation, which is administered for both the local and metastatic disease in children older than three years of age [9]. Yet, disease prognosis is still poor and CNS tumors of the childhood are, unfortunately, still manifesting high mortality rates and therapy resistance. Thus it is possible that improved treatments will come from the in-depth understanding of the tumor’s molecular machinery.

### 1.2. Gene Expression in CNS Tumors

There is overwhelming evidence on the role of gene expression and regulation in CNS tumors, both those of adults, as well of the children. miRNAs and mRNAs, have been studied thoroughly in the literature, where each has an abundant portion of literature dedicated to CNS tumors. A search in the principal literature databases returns more the 6000 articles on the topic of both miRNAs and mRNAs in CNS tumors.

### 1.3. Epigenetic Mechanisms in CNS Tumors

A very significant factor that has to be considered is the basic understanding of tumor biological mechanisms. In that sense, a very significant part of tumor mechanics is the epigenetic modification of the genome. Epigenetic mechanisms include the regulation of gene expression through methylation of genes or post-transcriptional modifications. Epigenetic mechanisms of biological systems has been neglected throughout time. Yet, it has been shown that they consist of a very significant regulatory mechanism.

#### 1.3.1. DNA Methylation and Cancer

The role of DNA methylation in cancer has been the topic of intensive study during the recent years. Since the discovery of DNA methylation mechanisms, a large part of the literature has reported that DNA methyltransferase aberrant activity is present in tumors [10,11,12,13,14,15]. It has been found that malignant cells often manifest increased total DNA methyltrasferase activity, significantly extensive loss of methylation, from otherwise physiologically methylated promoters, but also hypermethylation of normally unmethlylated DNA sites [16,17,18,19,20]. Several studies have reported the significance of gene promoter methylation. For example, the genes SPARC, UCHL1, NPTX2, PENK, and PDAC were investigated in pancreatic cancer, where they were found to be hypermethylated in patients with pancreatic cancer [21]. Similarly, studies have reported the presence of hypermethylated tumor suppressor gene’s promoters in lung cancer [22,23], gastric cancer [24], and breast cancer [25].

#### 1.3.2. DNA Methylation and CNS Tumors

Recent findings have highlighted the role of methylation on CNS tumors. In particular, several reports have shown that mutations of the isocitrate dehydrogenase 1 (IDH1) and IDH2, and H3 histone family member 3A, are strongly associated with DNA and histone methylation, with a frequent methylation aberration being the O6-methylguanine-DNA methyltransferase promoter in human diffuse gliomas [26]. Similarly, the promoter of MGMT has been shown to be hypermethylated in non-malignant tumors of the CNS [27]. In addition, a recent report showed that in glioblastomas the promoter of GBX2, PDGFRA, and GLI2 were hypermethylated and also linked to poor prognosis and resistance to chemotherapy [28]. In a recent study, the methylation of RASSF1 and CASP8 were reported as significant markers for the separation between childhood ependymoma and choroid plexus papilloma [29].

### 1.4. Patient Administration and Stratification Based on New Biomarkers

One very important and interesting aspect that arises from studies as the one presented here, is the potential use of molecular factors that could be used for patient prognosis, diagnosis, and most of all therapy. A quick search in the literature up-to-date, shows that there is a great interest in the scientific community for the role of miRNAs in CNS tumors. For example, in a very recent study it has been reported that the signatures of a miRNA set, was able to distinguish between primary CNS and non-malignant tumors. The highlight of this study, was that the distinction could be facilitated by miRNAs detected in the cerebrospinal fluid (CSF) [30]. The great advantage of miRNAs (but limited to) is that besides their role in tumor tissue biology, they are also found in circulation, being able to be detected more easily. Another interesting issue, outlined in the literature (on which we also agree) is that clinical advantages (prognosis, diagnosis, therapy) are not derived from a single miRNA, but rather from a cohort or repertoire of miRNAs. This brings about another very important aspect in tumor biology, which is its multi-factorial and therefore complex nature [31]. On the other hand, the most important aspect that has being discussed recently, is the possibility of therapeutic interventions using epigenetic mechanisms, such as miRNAs. Currently, there are no known miRNA therapies for human brain tumors. Yet, several studies have highlighted the use of these molecules in animal models. In particular, miR-370-3p [32], miR-142-3p [33], miR-181 [34], miR-124, miR-128, and miR-137 [35] are molecules that, when used in animal models, manifested potential anti-tumor and chemotherapy-enhancement properties.

Another interesting aspect is the type of approach in the study of CNS biology and the role of epigenetic regulatory mechanisms that would be whether investigate the tumor on the tissue level or plasma level. Both approaches are significant and provide significant information on the tumor’s biology. The investigation of miRNA levels in plasma/blood (thus circulating) could provide information for the prognostic and diagnostic course of the disease, while the investigation of epigenetic mechanisms on the tissue level provides information on the tumor’s biology along with the diagnostic and therapy-related knowledge. A quick search on the bibliographic databases shows that most studies are concerned with biology of the disease on the tumor’s site, while a smaller part is concerned with the circulating miRNA molecules. Yet, both approaches still remain quite significant for our understanding of the disease. From that perspective, we have chosen to investigate the biology of the tumors from the tissue approach, as we attempted to identify those miRNAs and epigenetic mechanisms that are common to the investigated tumors irrespective of their stratification and diversity.

### 1.5. Design and Aim of the Present Study

The current study, was performed on four levels; the first level included the diagnosis and staging of childhood CNS tumors through microscopy and immunohistochemical methods, the second level included the determination of miRNA expression levels using high throughput methodologies, the third level included the determination of mRNA levels using high throughput methodologies and the fourth level included the determination of the methylation status on specific genes. Our strategy consisted of two approaches; the first was to examine the relation of clinical variables and differential expressed genes and the second was to examine those miRNAs and mRNAs that were globally up- or down-regulated in all tumor samples. The present work is summarized in the flow chart of Figure S1.

Therefore, based on the aforementioned strategy, we aimed at determining the differential and common signatures of miRNA, mRNA expression and methylation in different childhood CNS tumors, in order to discover common regulatory mechanisms.

## 2. Materials and Methods

### 2.1. Patients and Tumor Samples

Overall, 49 surgically resected brain tumors from children diagnosed with a central nervous system (CNS) malignancy, were studied. In particular, tumors included: (a) pilocytic astrocytomas (PA) (*n* = 20); (b) ependymomas (EP) (*n* = 7); (c) medulloblastoma (*n* = 16); (d) atypical teratoid/rhabdoid tumors (ATRT) (*n* = 4); and (e) cortical dysplasia (CD) (*n* = 2); which were diagnosed according to the 2007 and 2016 WHO criteria [1,2,36,37]. As reference, healthy samples were used, obtained from deceased children (*n* = 13) as well as the “First-Choice Human Brain Reference RNA” (*n* = 1) (Ambion, Austin, TX, USA). The control group consisted of thirteen samples, who were dissected from deceased children, underwent autopsy and were diagnosed with no brain aberrancies. The specific anatomic sites obtained, included: cerebellum (*n* = 3), medulla oblongata (*n* = 3), parietal lobe (*n* = 3), and temporal lobe (*n* = 3), as previously described [3,4]. All tissues were immediately snap-frozen or processed after resection and stored a −80 °C until further processing. Patient characteristics are summarized in Table 1.

### 2.2. Diagnosis and Immunohistochemistry

#### 2.2.1. Diagnosis and Clinical Evaluation

Hematoxylin and eosin (H&E) staining and immunohistochemistry (IHC) for several markers, were performed for all brain tumor specimens, as we have previously reported [3,4,5]. All samples were snap-frozen after surgical resection and stored a −80 °C until use. Clinicopathologic information, such as age, tumor location, disease progression and survival for each specimen were collected by retrospective medical record review.

#### 2.2.2. Immunohistochemistry

Haematoxylin and eosin (H&E) staining and immunohistochemistry (IHC) has been previously reported [3,4,5]. In brief, dissected samples were tested for the expression of several markers, which included glial fibrillary acidic protein (GFAP) (*n* = 62), P38 (*n* = 52), neuromicrofilaments (NF) (*n* = 51), tumor protein p53 (p53) (*n* = 54), Bcl2 apoptosis regulator (BCL2) (*n* = 50), β-tubulin III (TUBB3) (*n* = 25), RNA binding Fox-1 homolog 3 (Neu-N) (*n* = 33), β-catenin (CTNNB1) (*n* = 16), pre-mRNA-splicing factor ini1 (INI1) (*n* = 21), marker of proliferation Ki-67 (MKI67) (*n* = 55), keratin (*n* = 34), mucin 1 cell surface associated (MUC1 or EMA) (*n* = 50), epidermal growth factor receptor (EGFR) (*n* = 16), P27 (*n* = 17), S100 calcium binding protein (S100) (*n* = 13), actin (*n* = 14), desmin (DES) (*n* = 15), and myelin (MBP) (*n* = 13). Each slide was individually evaluated and scored by two independent observers blinded to all clinical data. Discrepancies in scoring between the observers were resolved by additional review of the slides under a double headed microscope until a consensus was reached. For the evaluation we used a Nikon light microscope. The whole section was initially reviewed and representative areas were selected at low magnification (×100). Cell count was performed at high magnification (×400). The number of positive stained cells along with the total number of cells were counted in 10 different, non-overlapping fields per section. Then, the average of the cells was taken and the percentage of positive stained cells for each section was calculated (positive stained cells (%)). Clinicopathologic information such as age, tumor location, disease progression, and survival for each specimen were collected by retrospective medical record review.

### 2.3. RNA Extraction

Samples (*n* = 61) were processed for both total RNA, as well as miRNA extraction. In brief, total RNA and miRNAs were extracted using the Trizol standard protocol (Invitrogen, Carlsbad, CA, USA) and the mirVANA miRNA isolation kit (Ambion, Austin, TX, USA). The RNA quantity and quality were evaluated using a spectrophotometer (NanoDrop^®^ ND-1000 UV–vis, Nanogen Inc., San Diego, CA, USA), as previously described [3,4,5].

### 2.4. Microarray Profiling

#### 2.4.1. miRNA Profiling

MicroRNA profiling has been described previously in detail [3,4,5], which we reproduce in quotes as follows: “In brief, total RNA and miRNAs were extracted using the Trizol standard protocol (Invitrogen, Carlsbad, CA, USA) and the mirVANA miRNA isolation kit (Ambion, Austin, TX, USA). Labelling and hybridization were performed using the LabelIT miRNA labelling kit (Mirus Bio LLC, Madison, WI, USA) according to manufacturer’s instructions. Samples were hybridized to Applied MicroArrays (miRlink Bioarray 300054-3PK) platform. This array contained 1211 human miRNAs. Hybridization was performed at 37 °C with rotation at 145 rpm for 16 h. Images were scanned using Agilent Microarray Scanner (G2565CA) controlled by Agilent Scan Control 7.0 software. The total gene signals were extracted using the Imagene 6.0 software (Biodiscovery Inc., El Segundo, CA, USA) that contains summarized signal intensities for each miRNA by combining intensities of replicate probes and background subtraction” [3,4,5]. In total, 49 CNS tumor samples (the complete cohort described in Section 2.1.) and 13 control samples were investigated for their miRNA expressional profile. All microarray data are MIAME compliant.

#### 2.4.2. cRNA Profiling

Oligos microarray chips (~57k genes) were obtained from GE HealthCare (Chicago, IL, USA) and Applied Microarrays (Tempe, AZ, USA) (formerly Amersham Biosciences, Buckinghamshire, UK) (CodeLink 57k Human Whole Genome) [38,39,40]. Hybridization was performed with the CodeLink RNA amplification and labeling kit as described by the manufacturer, utilizing the Cy5 fluorescent dye. Slides were scanned with a microarray scanner (ScanArray 4000XL, Northville, MI, USA). Images were generated with ScanArray microarray acquisition software (GSI Lumonics, Northville, MI, USA). cRNAs from three experimental setups were used in single experiments with internal spikes as controls. The scanned images were further processed with the CodeLink Expression Analysis Software v5.0 from Amersham Biosciences (presently GE Health Care Inc., Chicago, IL, USA). The experimental setup was analyzed based on the reference-design, as described previously [41,42,43]. Gene expression values of tumor samples were compared against the mean value of the control samples. In total, 4 CNS tumor samples (one PA, one MB, one EP, and one ATRT) and 3 control samples were investigated for their mRNA expressional profile. All microarray data are MIAME compliant. This type of experimentation was performed in order to be used as a reference population for the applied ontological annotations in the present study. In other words, each ontological annotation analysis, requires a reference population of genes to be used, which can be used the complete genome or a certain gene cohort. In our case, we have used the DE genes detected from mRNA expression analysis as the reference gene cohort and this was used for any further bioinformatics analyses.

### 2.5. DNA Extraction

DNA was extracted from tumor samples, using the DNEasy Blood and Tissue kit (QIAGEN, Hilden, DE, Cat. Nr. 69504). PCR product was purified using the QIAquick Purification kit (QIAGEN, Hilden, DE, Cat. Nr. 28104).

### 2.6. Methylation

From the total patient cohort, children diagnosed with pilocytic astrocytomas (PA) (*n* = 13), ependymomas (EP) (*n* = 2), medulloblastoma (MB) (*n* = 8), and atypical teratoid rhabdoid tumor (ATRT) (*n* = 1) were evaluated for the possible methylation on specific genes. Twelve non-tumor brain samples were used (*n* = 12) as controls, as described in the previous section.

#### 2.6.1. Methylation-Specific MLPA (MS-MLPA)

Methylation-specific multiplex ligation-dependent probe amplification (MLPA) (MS-MLPA) is a semi-quantitative method for methylation profiling. In this study we used two MS-MLPA probe mixes (ME001-D1 tumor suppressor mix 1 and ME002-C1 tumor suppressor mix 2, MRC Holland (Amsterdam, NL) (www.mlpa.com, accessed 15 September 2021)) that contain methylation specific probes for 37 different tumor suppressor (TS) genes. Using the MS-MLPA technique we can distinguish the TS genes that keep the methylated status and therefore are silenced [44].

#### 2.6.2. Methylation-Specific PCR

For the validation of methylated genes, a methylation-specific PCR (MS-PCR) protocol was designed [45]. In particular, samples underwent complete bisulfate conversion using the EZ DNA Methylation-LightningTM kit (Zymo Research, Irvine, CA, USA) before being used in the MS-PCR according to the manufacturer’s instructions. The MS-PCR primers were designed using the MethPrimer software [46] and the PCR was performed at 55 °C. In particular, primers were designed for RASSF1 and CASP8 genes, in order to test more samples. Primer sets are summarized in Table 2.

### 2.7. Data Analysis

#### 2.7.1. Statistical Analysis

Continuous variables are expressed as mean ± standard deviation, unless indicated differently. Variable differences and association with clinical variables were conducted with the Kruskal–Wallis test. Patient’s characteristics are presented with absolute and relative frequencies (%). Chi-square test of independence was used to evaluate the association between having multiple methylated genes or aberrations and patients’ characteristics. The characteristics that were found statistically significant were entered in a logistic regression model in order to evaluate the probability of having multiple positive reactions. The level of statistical significance was set to a = 5%. For comparisons between groups, Student’s *t*-test and one-way analysis of variance (ANOVA) were performed for the continuous variables and Chi-square tests were used for the categorical variables. Post hoc comparisons (adjusted with Bonferroni criterion) were also performed when significant differences (*p* < 0.05) of the variables in ANOVA tests were identified. The characteristics that were found statistically significant were entered in a logistic regression model in order to evaluate the probability of having multiple positive reactions. The modeling of a quantitative variable based on one or more qualitative and quantitative parameters, was performed through linear regression. Multiple logistic regression was performed in order to evaluate the probability of having multiple positive reactions. The relative risk (RR), odds ratio (OR), absolute risk (AR) were calculated.

Methylation analysis and copy number variation (CNV) was performed with the Coffalyzer software (MRC Holland (www.mlpa.com, accessed 15 September 2021)). Indicative analysis results are presented in Figure S2. Statistical analysis has been performed with the Matlab^®^ computational environment (The Mathworks, Inc., The Natick, MA, USA).

#### 2.7.2. Microarray Data Analysis

##### Microarray Data Pre-Processing

The extracted microarray data were entered into a Microsoft Excel^®^ file.

##### Microarray Data Post-Processing

Microarray data were initially background-corrected using the multiplicative background correction (MBC) approach [47]. In particular, MBC subtracts the logarithmic estimates of the background intensity from the logarithmic foreground intensity. After background correction, negative values were removed and replaced with “NaN” values. Our intention was to find miRNA and mRNA expression, even those of low expression values. It is possible that not only those values that are of great difference are of importance, but also those that have very low expression values could be of biological importance. Microarray data post-processing has been performed with the Matlab^®^ computational environment (The Mathworks, Inc., The Natick, MA USA)

##### Microarray Data Normalization

Microarray data normalization was then performed using three algorithms; (a) Loess [48], (b) rank invariant, and (c) quantile algorithm. To account for differences across series we used the log2-transformed ratio:

(1) $$R_{i,j}=\log_{2}\left(\frac{x_{i,j}}{{\bar{x}}_{total}}\right)\;X_{i,j}=2^{R_{i.j}}$$

where, *R_i,j_* is the global mean-transformed ratio, *x_i,j_* is the expression value of gene *i* and sample *j*, *X_i,j_* is the restructured value of the *i*th gene and *j*th sample.

The three algorithms were compared for their efficiencies. In general, the quantile algorithm performed better as comparted to the other two. Microarray data normalization has been performed with the Matlab^®^ computational environment (The Mathworks, Inc., The Natick, MA, USA).

##### Elimination of Duplicate Transcripts

To reduce the complexity of the dataset, we followed the replicate averaging approach proposed by Uzman et al. [49]. Calculations of duplicate genes was performed with the Matlab^®^ computational environment (The Mathworks, Inc., The Natick, MA, USA).

##### Detection of Differentially Expressed Genes (DEGs)

We used the Student’s *t*-test [50] to identify the differentially expressed miRNA and mRNA genes (DE miRNAs, DE mRNAs) across all tumor samples and compared them to all control samples. The false discovery rate (FDR) was calculated, as previously described [51,52,53]. The DE miRNA and mRNA genes per experiment were identified at a confidence level of 95%. DE miRNAs and mRNAs were treated in two different ways. Data were further processed and analyzed as “ratios”, i.e., as gene expression values calculated as the log2-transformed ratio of each tumor sample over the mean of all control samples, using the following formula:

(2) $$E=\log_{2}\left(\frac{F_{Tumor,i,j}}{{\bar{F}}_{Controls,j}}\right)$$

where *E* is the expression value, *F_Tumor,i,j_* is the expression value of tumor sample *i* and miRNA/mRNA *j*, ${\bar{F}}_{Controls,j}$ is the mean expression value of all controls and miRNA/mRNA *j*. In addition, data were also analyzed as “naturals”, meaning DEGs that are non-log2-transformed and thus including the control and tumor samples separately. DEGs have been estimated with the Matlab^®^ computational environment (The Mathworks, Inc., The Natick, MA, USA).

##### Unsupervised Classification Methods

DEGs were further analyzed for common expression patterns using classification methods. To gain further insight into the gene expression data, we used unsupervised hierarchical clustering (HCL) and k-means classification [54,55]. HCL with dendrogram, was used and correlations were calculated with Euclidean distance. K-means classification [54,55] was recently reported as one of the best performing clustering approaches for microarray class discovery studies [56]. We applied the squared Euclidean as a distance measure, since it is generally considered to be a more appropriate measure for use with k-means and found to outperform for ratio-based measurements [57]. We used 100 iterations and the optimal cluster number for the k-means algorithm was estimated using the Calinski–Harabasz criterion. Complete k-means clusters, centroids, and sorted centroids [58] were utilized. The DE miRNAs/mRNAs, were also classified based on their diagnosis categorical variable. In particular, the mean values of all samples with respect to the diagnosis was estimated and the resulting descriptive statistical measure was utilized for further k-means classification. Gene expression was also analyzed with respect to the chromosomal distribution of the DE miRNAs/mRNAs. We explored the mean expression per chromosome and heat-maps of chromosomal-related expression. Unsupervised classification methods have been performed with the Matlab^®^ computational environment (The Mathworks, Inc., The Natick, MA, USA).

##### Unsupervised Classification Methods: Descriptive K-Means

In order to further investigate the expression patterns of DE miRNAs, we have used a variation of the k-means algorithm, where instead of using the complete sample cohort, i.e., each sample separately, we used the categorical properties of the sample cohort. In the classical k-means algorithm a matrix of *m* × *n* is used, where *m* is the number of genes/miRNAs and *n* is the number of samples. In our variation, a new matrix is formed with dimensions *m* × *q*, where *q* is the number of unique values of a categorical variable. For example, in the present cohort 49 tumor samples were utilized and could be divided into five distinct diagnostic categories (e.g., ependymoma, astrocytoma, medulloblastoma etc.) and, hence, *q* = 5. K-means clustering was implemented with the same parameterization as the aforementioned typical k-means approach.

##### Common Expression Patterns in DE miRNAs/mRNAs

DE miRNAs/mRNAs were examined for possible common expression patterns, i.e., miRNAs/mRNAs that were either down- or up-regulated in all CNS tumor samples, irrespective of the tumor diagnosis. The clusters revealed by unsupervised classification were examined separately. Each miRNA was counted for its occurrences for up- or down-regulation in all samples and the result was divided by the total number of samples, giving the percentage of up- or down-regulated samples of the respective miRNA/mRNA. We have looked for miRNAs/mRNAs that were either up or down-regulated in all samples (100%), in 90–99% of all samples, 80–89% of all samples, and 75–80% of all samples.

##### Gene Ontology (GO) Enrichment Analysis

We performed GO enrichment analysis using the gprofiler [59], and WebGestalt web-tools [60]. Relations of the differentially expressed genes and the transcription factor binding motifs were further investigated using the Pubgene Ontology Database (www.pubgene.org, accessed 12 May 2020). Gene definitions and functions were based on the National Institute of Health databases (http://www.ncbi.nlm.nih.gov/sites/entrez/, accessed 27 September 2020).

##### Pathway Analysis

Pathway analysis was performed using the gprofiler [59] and WebGestalt web-tools [60].

##### miRNA Enrichment Analysis

Enriched miRNA targets were identified using the miTEA web-tool [61,62], as well as for miRNA enrichment analysis the MiEEA [63], and TAM 2.0 [64,65], web-tools were used.

#### 2.7.3. Receiver Operating Characteristic (ROC) Analysis

ROC curves and naïve-Bayes classification were used to investigate the diagnostic ability of the co-deregulated miRNAs/mRNAs between CNS tumors and control samples. In the case of naïve-Bayes classification, the algorithm used the Bayes theorem, and (naïvely) assumes that the predictors are conditionally independent, given a class. Naïve Bayes classifiers assign observations to the most probable class (in other words, the maximum a posteriori decision rule). ROC analysis has been performed with the Matlab^®^ computational environment (The Mathworks, Inc., The Natick, MA, USA).

### 2.8. Ethics Statements

All experiments were conducted in compliance with the international biomedical studies stipulations, with reference to the Declaration of Helsinki of the World Medical Association. No personal data of patients were kept, while it was impossible to trace back any personal data from the data collected for the present study. Informed written consent was obtained from the parents of all children included in the study. The present study was conducted with the approval of “Aghia Sophia” Children’s Hospital Ethics Committee (Protocol No. 35/19.16/09/13).

## 3. Results

In the present study, we have analyzed a cohort of pediatric patients suffering from different types of CNS tumors, including ependymoma, medulloblastoma, ATRT, and astrocytoma.

### 3.1. Patients and Tumor Samples

Our patient cohort included 39 males and 25 females with ages mean 6.59 ± 4.59 years, median 6.20 (min 0.03, max 16.06 years). The mean age of PA patients was 5.86 ± 3.42 years with a male–female ratio of 1:1, the median age for the EP patients was 5.13 ± 5.40 years with a male–female ratio of 6:1, the mean age for the MB patients was 6.92 ± 4.70 years with a male–female ratio of 1:1, the ATRT mean age was 2.46 ± 3.54, while the non-malignant cohort was 9 years consisted of males only (Table 1). Among the patients’ cohort, 24 patients (85.7%) remained in complete remission (after therapy); 17 PA, 5 EP patients, and 2 CD patients, and 4 patients (14.3%) succumbed from the disease; 2 PA patients and 2 EP patients. The laboratory and clinical parameters of all patients were examined in relation to the miRNA/mRNA profiles obtained. For part of the patient cohort, the information concerning the time of death was not known and, thus, it was not possible to calculate the overall survival for all patients.

### 3.2. miRNA and mRNA Expression

**Figure 1:**
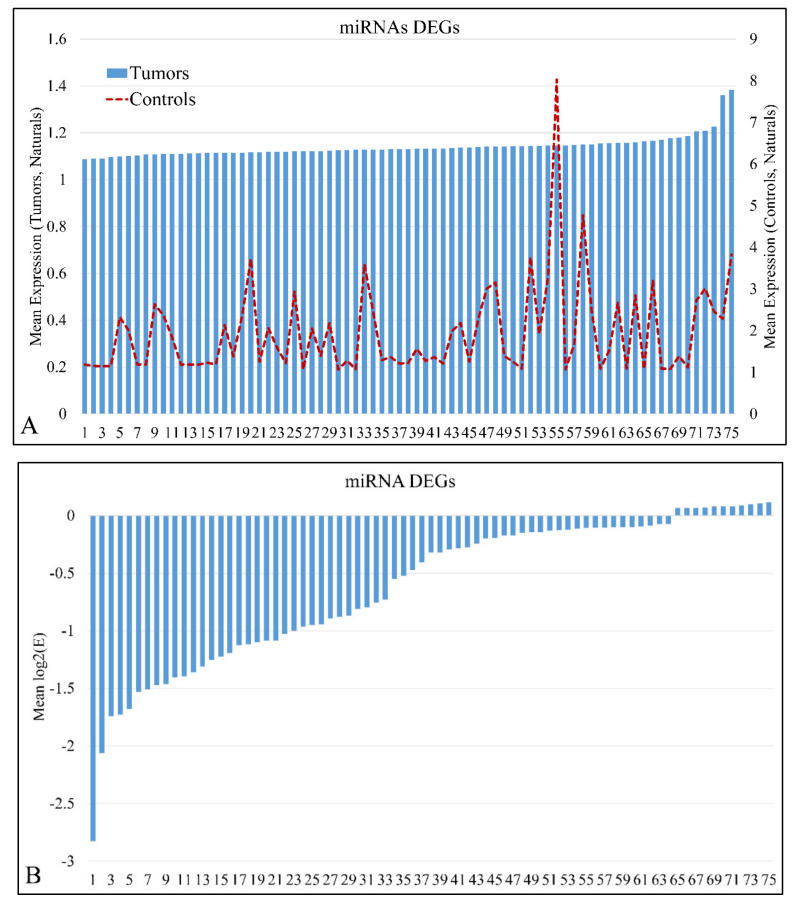
Mean expression of DE miRNAs in ascending order. The miRNAs naturals presented are in the following order (from 1 to 75): hsa-miR-3655, hsa-miR-128, hsa-miR-520a-3p, hsa-miR-3180-3p, hsa-miR-214, hsa-miR-1182, hsa-miR-580, hsa-miR-1204, hsa-miR-3622b-5p, hsa-miR-9*, hsa-miR-491-3p, hsa-miR-770-5p, hsa-miR-490-3p, hsa-miR-183, hsa-miR-34c-3p, hsa-miR-4276, hsa-miR-2909, hsa-miR-194*, hsa-miR-3191, hsa-miR-30e*, hsa-miR-135a*, hsa-miR-3672, hsa-miR-25*, hsa-miR-489, hsa-miR-188-5p, hsa-miR-34a, hsa-miR-606, hsa-miR-612, hsa-miR-514b-5p, hsa-miR-600, hsa-miR-765, hsa-miR-320e, hsa-miR-3675-5p, hsa-miR-876-5p, hsa-miR-643, hsa-miR-4261, hsa-miR-410, hsa-miR-548y, hsa-miR-4307, hsa-miR-526b*, hsa-miR-19b, hsa-miR-3614-5p, hsa-miR-4251, hsa-miR-3618, hsa-miR-106b*, hsa-miR-4270, hsa-miR-542-3p, hsa-miR-96*, hsa-miR-4317, hsa-miR-576-5p, hsa-miR-4267, hsa-miR-3202, hsa-miR-584, hsa-miR-645, hsa-miR-592, hsa-miR-226, hsa-miR-4329, hsa-miR-656, hsa-miR-3616-3p, hsa-miR-1246, hsa-miR-214*, hsa-miR-3942, hsa-miR-1234, hsa-miR-302b, hsa-miR-149*, hsa-miR-23b, hsa-miR-125b, hsa-miR-147, hsa-miR-3123, hsa-miR-122*, hsa-miR-4330, hsa-miR-95, hsa-miR-582-5p, hsa-miR-130b, hsa-miR-649 (**A**). The miRNAs ratios presented are in the following order (from 1 to 75): hsa-miR-649, hsa-miR-130b, hsa-miR-4330, hsa-miR-95, hsa-miR-122*, hsa-miR-3123, hsa-miR-582-5p, hsa-miR-23b, hsa-miR-147, hsa-miR-302b, hsa-miR-214*, hsa-miR-125b, hsa-miR-3942, hsa-miR-656, hsa-miR-3616-3p, hsa-miR-1234, hsa-miR-1226, hsa-miR-4329, hsa-miR-645, hsa-miR-592, hsa-miR-3202, hsa-miR-1246, hsa-miR-584, hsa-miR-576-5p, hsa-miR-4267, hsa-miR-4317, hsa-miR-542-3p, hsa-miR-96*, hsa-miR-3618, hsa-miR-4270, hsa-miR-149*, hsa-miR-106b*, hsa-miR-4251, hsa-miR-3614-5p, hsa-miR-548y, hsa-miR-4307, hsa-miR-19b, hsa-miR-3675-5p, hsa-miR-876-5p, hsa-miR-410, hsa-miR-643, hsa-miR-4261, hsa-miR-526b*, hsa-miR-600, hsa-miR-606, hsa-miR-612, hsa-miR-135a*, hsa-miR-514b-5p, hsa-miR-4276, hsa-miR-765, hsa-miR-194*, hsa-miR-3180-3p, hsa-miR-25*, hsa-miR-1204, hsa-miR-3672, hsa-miR-34c-3p, hsa-miR-9*, hsa-miR-3622b-5p, hsa-miR-489, hsa-miR-491-3p, hsa-miR-770-5p, hsa-miR-128, hsa-miR-3655, hsa-miR-520a-3p, hsa-miR-214, hsa-miR-183, hsa-miR-580, hsa-miR-1182, hsa-miR-320e, hsa-miR-3191, hsa-miR-2909, hsa-miR-490-3p, hsa-miR-188-5p, hsa-miR-30e*, hsa-miR-34a (**B**).

miRNA expression levels of all samples (*n* = 61) were estimated and were sorted in ascending order in order to identify expression patterns of DE miRNAs. In total, 75 miRNAs were detected as DEGs, with a FDR < 0.05 at *p* < 0.05 (Table S1). In addition, 869 mRNAs were detected as DEGs with a FDR < 0.01 at *p* < 0.05. DE mRNAs were used further on, in the present analysis, in order to identify miRNA targets with relation to the mRNA expression profile. Mean miRNA DEGs naturals in tumor and control samples separately are presented in Figure 1A, while miRNA ratios of tumors over controls are presented in Figure 1B.

### 3.3. miRNA Differential Expression with Respect to Clinical Parameters

#### 3.3.1. miRNA Expression Profiling with Respect to Gender

**Figure 2:**
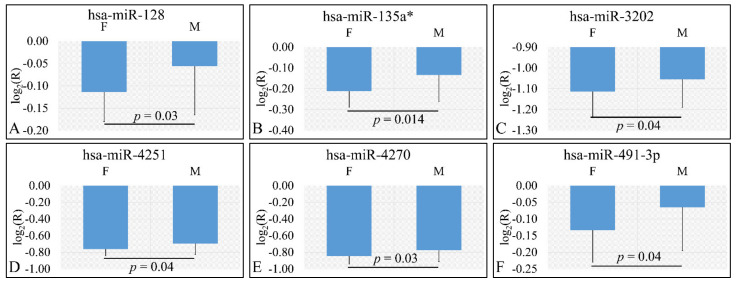
DE miRNAs with respect to gender (*n_males_* = 39, *n_females_* = 25). DE miRNAs included miR-128 (**A**), miR-135a* (**B**), miR-3202 (**C**), miR-4251 (**D**), miR-4270 (**E**), miR-491-3p (**F**). All miRNAs presented include those that are significantly different between male and female children (Legend: DE: Differentially expressed, R: Relative expression, where $R=\frac{E_{tumors}}{E_{controls}}$ and *E* is the expression levels of controls and tumors respectively, M: Male, F: Female).

Four miRNAs were found to be DE with respect to gender. In particular DE miRNAs included miR-128 (*p* = 0.03) (Figure 2A), miR-135a* (*p* = 0.014) (Figure 2B), miR-3202 (*p* = 0.04) (Figure 2C), miR-4251 (*p* = 0.04) (Figure 2D), miR-4270 (*p* = 0.03) (Figure 2E), and miR-491-3p (*p* = 0.04) (Figure 2F). Interestingly, all six miRNAs were found to be up-regulated in female patients as compared to male patients. In the case of gender, we have estimated the ratios of gene expression and not the “natural” values due to the fact that gender “natural” values included both control and tumor values.

#### 3.3.2. miRNA Expression Profiling with Respect to Gender

**Figure 3:**
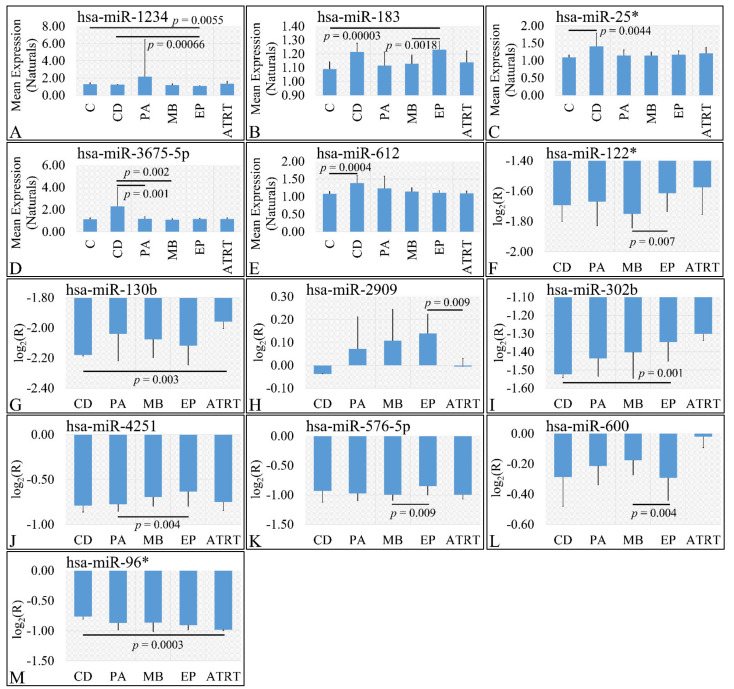
DE miRNAs with respect to diagnosis. Significant differences were estimated using ANOVA with Bonferroni correction. Significant differences were observed between cortical dysplasia (*n* = 2) and ependymoma (*n* = 7), as well as controls (*n* = 13) and ependymomas (*n* = 7) with respect to miR-1234 (**A**). Significant differences were observed between controls and ependymoma (*n* = 7), as well as medulloblastoma (*n* = 16) and ependymomas (*n* = 7) with respect to miR-183 (**B**). Significant differences were observed between controls (*n* = 13) and cortical dysplasias (*n* = 2) with respect to miR-25* (**C**). Significant differences were observed between cortical dysplasia (*n* = 2) and pilocytic astrocytomas (*n* = 20), as well as cortical dysplasias (*n* = 2) and medulloblastomas (*n* = 16) with respect to miR-3675-5p (**D**). Significant differences were observed between controls and cortical dysplasias (*n* = 2) with respect to miR-612 (**E**). When including control samples, calculating the expression ratios significant differences were observed between medulloblastomas and ependymomas (*n* = 7) with respect to miR-122* (**F**). Significant differences were observed between cortical dysplasia (*n* = 2) and ATRT (*n* = 4) with respect to miR-130b (**G**). Significant differences were observed between ependymomas (*n* = 7) and ATRTs (*n* = 4) with respect to miR-2909 (**H**). Significant differences were observed between cortical dysplasias (*n* = 2) and ependymomas (*n* = 7) with respect to miR-302b (**I**). Significant differences were observed between pilocytic astrocytomas (*n* = 20) and ependymomas (*n* = 7) with respect to miR-4251 (**J**). Significant differences were observed between medulloblastomas (*n* = 16) and ependymomas with respect to miR-576-5p (**K**). Significant differences were observed between medulloblastomas (*n* = 16) and ependymomas (*n* = 7) with respect to miR-600 (**L**) and finally significant differences were observed between cortical dysplasias and ATRT with respect to miR-96* (**M**) (Legend: DE: Differentially expressed, R: Relative expression, where $R=\frac{E_{tumors}}{E_{controls}}$ and *E* is the expression levels of controls and tumors respectively, C: Controls, CD: Cortical dysplasia, PA: Pilocytic astrocytoma, MB: Medulloblastoma, EP: Ependymoma, ATRT: Atypical teratoid rhabdoid tumor).

In the investigation of diagnosis, we have used both the “natural” values, as well as ratios of gene expression. Significant differences were observed between cortical dysplasia and ependymoma (*p* = 0.00066), as well as controls and ependymomas (*p* = 0.0055) with respect to miR-1234 (Figure 3A). In addition, significant differences were observed between controls and ependymoma (*p* = 0.00003), as well as medulloblastoma and ependymomas (*p* = 0.0018) with respect to miR-183 (Figure 3B). Similarly, significant differences were observed between controls and cortical dysplasias (*p* = 0.0044) with respect to miR-25* (Figure 3C). Significant differences were observed between cortical dysplasia and pilocytic astrocytomas (*p* = 0.001), as well as cortical dysplasias and medulloblastomas (*p* = 0.002) with respect to miR-3675-5p (Figure 3D), whereas miRNA expression manifested also a descending pattern from cortical dysplasias to ATRT (Figure 3D). Further on, significant differences were observed between controls and cortical dysplasias (*p* = 0.0004) with respect to miR-612 (Figure 3E) with a similar descending pattern as in the case of miR-3675-5p. By including control samples and calculating the expression ratios, significant differences were observed between medulloblastomas and ependymomas (*p* = 0.007) with respect to miR-122* (Figure 3F), between cortical dysplasia and ATRT (*p* = 0.004) with respect to miR-130b (Figure 3G), ependymomas and ATRTs (*p* = 0.0009) with respect to miR-2909 (Figure 3H). Interestingly, significant differences were observed between cortical dysplasias and ependymomas (*p* = 0.001) with respect to miR-302b (Figure 3I), with an ascending pattern as we move from cortical dysplasias to ATRTs, indicating a possible relation to tumor aggressiveness. Subsequently, significant differences were observed between pilocytic astrocytomas and ependymomas (*p* = 0.004) with respect to miR-4251 (Figure 3J), between medulloblastomas and ependymomas (*p* = 0.009) with respect to miR-576-5p (Figure 3K), between medulloblastomas and ependymomas (*p* = 0.004) with respect to miR-600 (Figure 3L), and, finally, between cortical dysplasias and ATRT (*p* = 0.0003) with respect to miR-96* (Figure 3M).

#### 3.3.3. miRNA Expression Profiling with Respect to Tumor Grade

**Figure 4:**
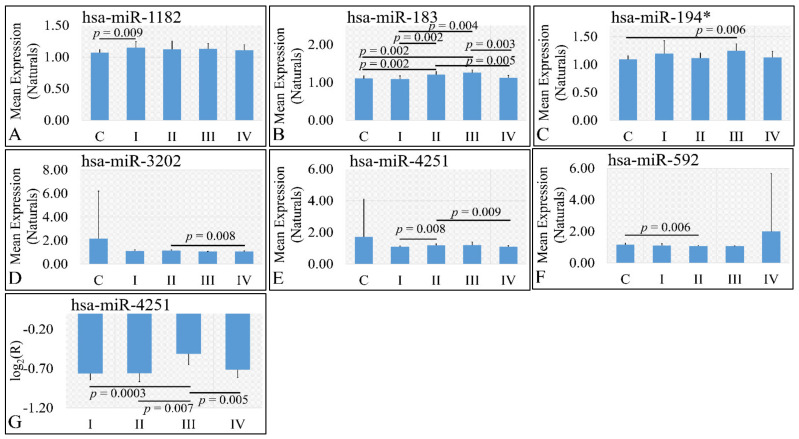
DE miRNAs with respect to tumor grade. Significant differences were estimated using ANOVA with Bonferroni correction. Significant differences were observed between controls and grade I (*n* = 16) tumors (*p* = 0.009) with respect to miR-1182 (**A**). Significant differences were observed between controls and grade II tumors (*n* = 9) (*p* = 0.002), between controls and grade III tumors (*n* = 3) (*p* = 0.002), between grade I (*n* = 16) and grade III (*n* = 3) tumors (*p* = 0.004), between grade II (*n* = 9) and grade IV (*n* = 18) tumors (*p* = 0.005), as well as between grade III (*n* = 3) and grade IV (*n* = 18) tumors (*p* = 0.005) with respect to miR-183 (**B**). Significant differences were observed between controls and grade III (*n* = 3) tumors (*p* = 0.006) with respect to miR-194* (**C**). Significant differences were observed between grade II (*n* = 9) and grade III (*n* = 3) tumors (*p* = 0.008) with respect to miR-3202 (**D**). Significant differences were observed between grade I (*n* = 16) and grade II tumors (*n* = 9) (*p* = 0.008), as well as between grade II (*n* = 9) and grade IV (*n* = 18) tumors (*p* = 0.009) with respect to miR-4251 (**E**). Significant differences were observed between controls and grade II (*n* = 9) tumors (*p* = 0.006) with respect to miR-592 (**F**). Finally, when including control samples, calculating the expression ratios significant differences were observed between grade I (*n* = 16) and grade III tumors (*n* = 3) (*p* = 0.0003), between grade II (*n* = 9) and grade III (*n* = 3) tumors (*p* = 0.007), as well as between grade III (*n* = 3) and grade IV (*n* = 18) tumors (*p* = 0.005) with respect to miR-4251 (**G**) (Legend: R: Relative expression, where $R=\frac{E_{tumors}}{E_{controls}}$ and *E* is the expression levels of controls and tumors, respectively, C: Controls).

Six miRNAs were found to be DE with respect to tumor grade. In particular, miR-1182 was found to be DE between controls and grade I tumors (*p* = 0.009) (Figure 4A). Significant differences were also observed between controls and grade II tumors (*p* = 0.002), between controls and grade III tumors (*p* = 0.002), between grade I and grade III tumors (*p* = 0.004), between grade II and grade IV tumors (*p* = 0.005), as well as between grade III and grade IV tumors (*p* = 0.005) with respect to miR-183 (Figure 4B), as well as significant differences were observed between controls and grade III tumors (*p* = 0.006) with respect to miR-194* (Figure 4C). miR-3202 manifested a descending pattern from controls to grade IV tumors with significant differences between grade II and grade III tumors (*p* = 0.008) (Figure 4D). Further on, significant differences were observed between grade I and grade II tumors (*p* = 0.008), as well as between grade II and grade IV tumors (*p* = 0.009) with respect to miR-4251 (Figure 4E) and between controls and grade II tumors (*p* = 0.006) with respect to miR-592 (Figure 4F). Finally, when including control samples, calculating the expression ratios significant differences were observed between grade I and grade III tumors (*p* = 0.0003), between grade II and grade III tumors (*p* = 0.007), as well as between grade III and grade IV tumors (*p* = 0.005) with respect to miR-4251 (Figure 4G).

#### 3.3.4. miRNA Expression Profiling with Respect to Protein Markers

**Figure 5:**
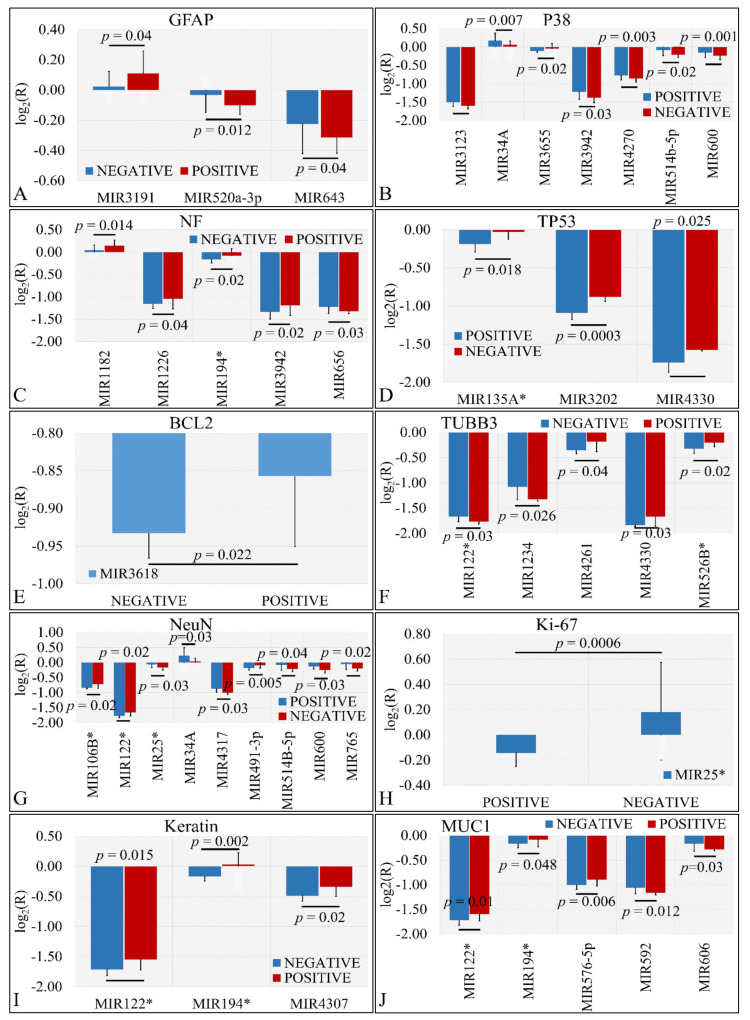
DE miRNAs with respect to protein markers. Significant differences were observed between tumor samples with positive or negative protein markers. In particular, significant differences were observed with respect to glial fibrillary acidic protein (GFAP) (**A**), P38 (**B**), Neuromicrofilaments (NF) (**C**), Tumor protein p53 (TP53) (**D**), Bcl2 apoptosis regulator (BCL2) (**E**), β-tubulin III (TUBB3) (**F**), RNA binding Fox-1 homolog 3 (NeuN) (**G**), Marker of proliferation Ki-67 (Ki-67) (**H**), Keratin (**I**), and Mucin 1 cell surface associated (MUC1) (**J**) (Legend: R: Relative expression, where $R=\frac{E_{tumors}}{E_{controls}}$ and *E* is the expression levels of controls and tumors, respectively).

Several miRNAs were identified to present significant differences with respect to the presence of protein markers in tumors. In particular, we have found that miR-3191, miR-520a-3p and miR-643 were significantly different with respect to GFAP expression (Figure 5A). Similarly, miR-3123, miR-34a, miR-3655, miR-3942, miR-4270, miR-514b-5p, and miR-600 manifested significant differences with respect to P38 expression (Figure 5B). Further on, miR-1182, miR-1226, miR-194*, miR-3942, and miR-656 showed significantly different expression levels with respect to NF expression (Figure 5C). Interestingly, miR-135a*, miR-3202, and miR-4330 were significantly down-regulated in TP53 negative tumors as compared to TP53 positive tumors (Figure 5D). In the case of BCL2, miR-3618 manifested higher expression levels in BCL2 positive levels as compared to BCL2 negative tumors (Figure 5E). Further on, miR-122*, miR-1234, miR-4261, miR-4330, and miR-526b* manifested significant differences in TUBB3 positive and negative tumors (Figure 5F). The relation of NeuN expression manifested the largest number of miRNAs with significant differences. In particular, mIR-106b*, miR-122*, miR-25*, miR-34a, miR-4317, miR-491-3p, miR-5143-5p, miR-600 and miR-765 presented significant differences with respect to TUBB3 expression (Figure 5G). As in the case of BCL2, only one miRNA manifested significant difference with respect to Ki-67 expression. In particular, miR-25* manifested higher expression levels in Ki-67 negative tumors as compared to Ki-67 positive levels (Figure 5H). In the case of keratin, miR-122*, miR-194* and miR-4307 manifested higher expression levels in keratin-positive tumors as compared to keratin-negative tumors (Figure 5I). Finally, miR-122*, miR-194*, miR-576-5p, miR-592 and miR-606 were differentially expressed in MUC1-positive tumors as compared to MUC1-negative tumors (Figure 5J).

### 3.4. Methylation

Part of our analysis included the investigation of the promoter methylation of a set of tumor suppressor and apoptotic genes. The gene repertoire examined included the promoters of CASP8, RASSF1, MGMT, MSH6, GATA5, ATM1, TP53, CADM1, and RB1 genes.

#### 3.4.1. Descriptive and Association Statistics of Tumors

##### Relations to Methylation

Tumor samples manifested a methylation pattern. In particular, methylated apoptotic genes included CASP8, RASSF1, MGMT, MSH6, GATA5, ATM1, TP53, CADM1, and RB1. Methylation was detected in one target gene, yet in some cases more than one genes were found to be simultaneously methylated. More specifically, it appeared that apoptotic gene methylation was significantly dependent on the samples’ status that is if the sample was a neoplasm or a control (*χ^2^* = 16.19, *p* = 0.040). Similar dependence was observed with respect to malignant, benign tumors and controls (*χ^2^* = 30.90, *p* = 0.014), as well as with respect to MB, PA, ATRT, EP, and control samples (*χ^2^* = 65.86, *p* = 0.0004). Further on, similar dependence was manifested for tumor grade (*χ^2^* = 55.55, *p* = 0.0061). Interestingly, it appeared that there was no significant dependence observed with a particular methylated gene, but rather with the simultaneous presence of all detected methylated genes. The results of this analysis are summarized in Table 3.

##### Relations to Methylation

As in the case of gene methylation we have examined dependence of clinical parameters with respect to CNV. There were no significance dependence observed with any of the observed CNVs, which included duplication of CD27, deletion of CD6, deletion of CD6/CDKN2A/GATA5, and CD44 duplication. The results are summarized in Table 4.

#### 3.4.2. Statistics of Total Number of Methylated Genes

**Figure 6:**
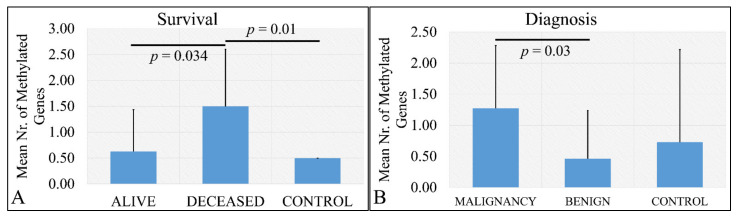
Number of methylated genes with respect to survival (**A**) and diagnosis (**B**). Analysis of the correlation of gene methylation to clinical characteristics patients have. In particular, it appeared that there is a significant difference in the number of methylated genes between deceased patients and control samples, as well as alive patients and control samples (**A**) and between malignant and benign tumors (**B**).

Deceased patients (meaning that the patient did not survive at the time of the study) had significantly more methylated genes as compared to living patients, as well as deceased patients has more methylated genes as compared to control samples (Figure 6A). The aforementioned observations were confirmed by the finding that there was a significant difference in the total number of methylated genes with respect to the tumor type that is if the tumor was malignant or benign and control samples. In particular, it appeared that malignant tumor types had significantly more methylated genes as compared to benign tumor types (Figure 6B). No significance was observed between malignant tumors and control samples.

#### 3.4.3. Methylation-Specific PCR

**Figure 7:**
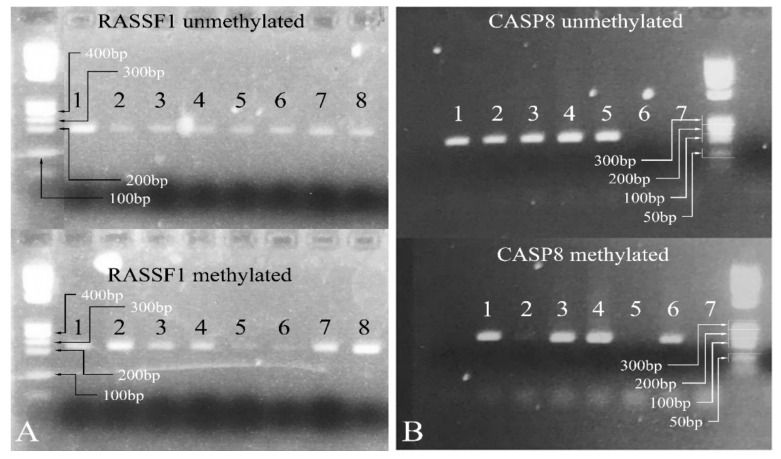
Methylation-specific PCR. In the case of RASSF1, all samples show both methylated and unmethylated alleles (**A**). Similarly, for CASP8, samples 1, 3, and 4 showed both methylated and unmethylated alleles (**B**). Samples 2 and 5 were 100% unmethylated and sample 6 was 100% methylated (Samples: in (**A**) 1: PA; 2: PA; 3: PA; 4: MB; 5: PA; 6: PA; 7: PA; 8: PA. Samples: in (**B**) 1: PA; 2: PA; 3: PA; 4: PA; 5: MB; 6: PA; 7: negative control) (Note: the samples presented in the gels presenting the methylated and unmethylated genes are not the same. For example, sample 1 in the RASSF1 unmethylated gel is not the same with sample 1 in the RASSF1 methylated gel as they correspond to two different patients, although they are both PA. The same holds true for the samples investigated for the CASP8 gene. Legend: PA: Pilocytic astrocytoma, MB: Medulloblastoma. Note: Samples in (**A**) were electrophoresed on the same agarose gel).

Since the most significant signal was obtained for the genes *CASP8* and *RASSF1* in order to test more samples, a methylation specific PCR (MS-PCR) protocol was designed [45]. The MS-PCR primers were designed using the MethPrimer software [46], and the PCR was performed at 55 °C. Representative results are shown in Figure 7.

#### 3.4.4. miRNA Differential Expression with Respect to Methylated Genes

**Figure 8:**
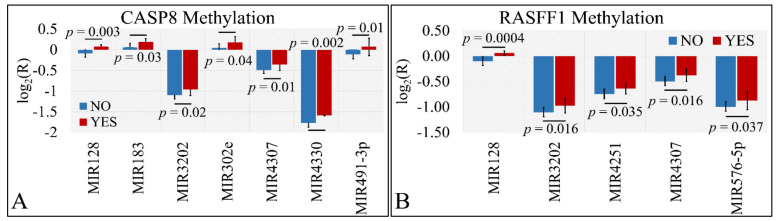
DE miRNAs with respect to gene methylation. Significant differences were observed between tumor samples with methylated and un-methylated genes. In particular, miR-128, miR-183, miR-3202, miR-302e, miR-4307, miR-4330, and miR-491-5p manifested significant differences with respect to CASP8 methylation (**A**). Similarly, miR-128, miR-3202, miR-4251, miR-4307, and miR-576-5p manifested significant differences with respect to RASFF1 methylation (**B**) (Legend: R: Relative expression, where $R=\frac{E_{tumors}}{E_{controls}}$ and *E* is the expression levels of controls and tumors, respectively, YES: methylated gene, NO: un-methylated gene).

Differential expression was also examined with respect to gene methylation. In particular, we have tested the presence of significant differences with respect to the methylation status of CASP8 and RASFF1 genes. In particular, miR-128, miR-183, miR-3202, miR-302e, miR-4307, miR-4330, and miR-491-5p manifested significant differences with respect to CASP8 methylation (Figure 8A). Similarly, miR-128, miR-3202, miR-4251, miR-4307, and miR-576-5p manifested significant differences with respect to RASFF1 methylation (Figure 8B).

### 3.5. Hierarchical Clustering (HCL)

**Figure 9:**
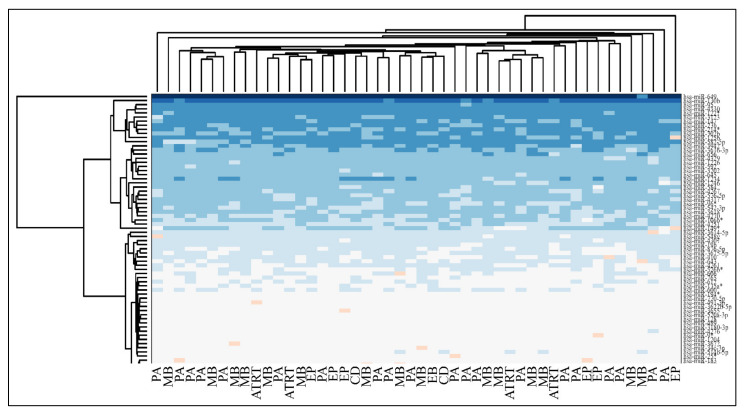
Hierarchical clustering (HCL) of miRNA expression with respect to diagnosis (Legend: PA: Pilocytic Astrocytoma, MB: Medulloblastoma, EP: Ependymoma, CD: Cortical Dysplasia, ATRT: atypical teratoid/rhabdoid tumors).

Unsupervised two-way hierarchical clustering (HCL) with Euclidian distance did not discriminate accurately between all tumor types and the normal control group, as well as between PAs and controls or EPs and controls (Figure 9).

### 3.6. Chromosomal Distribution of DE miRNAs

**Figure 10:**
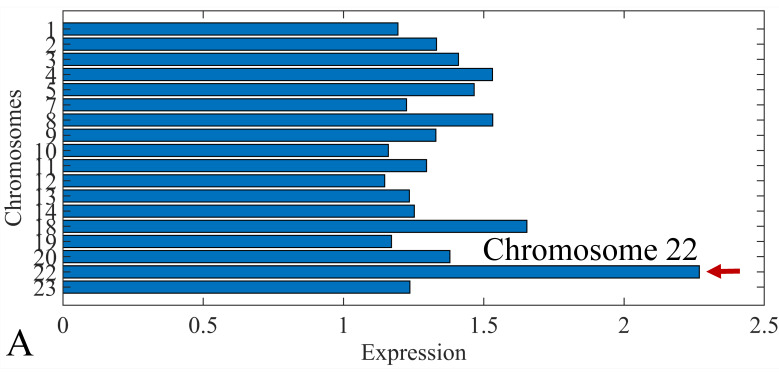

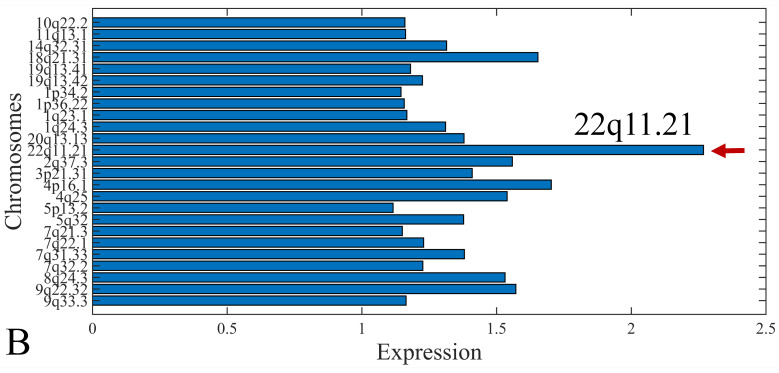

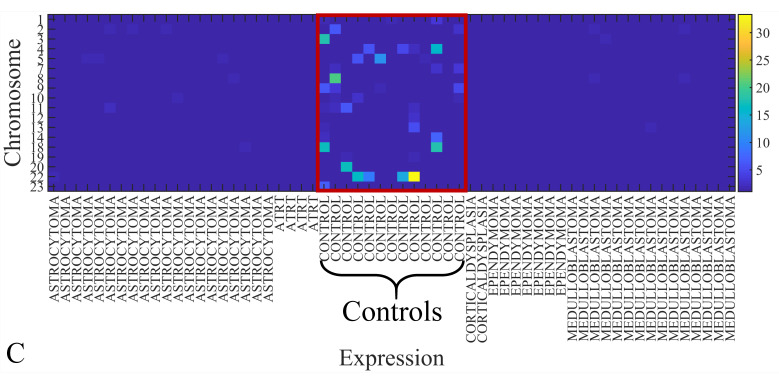

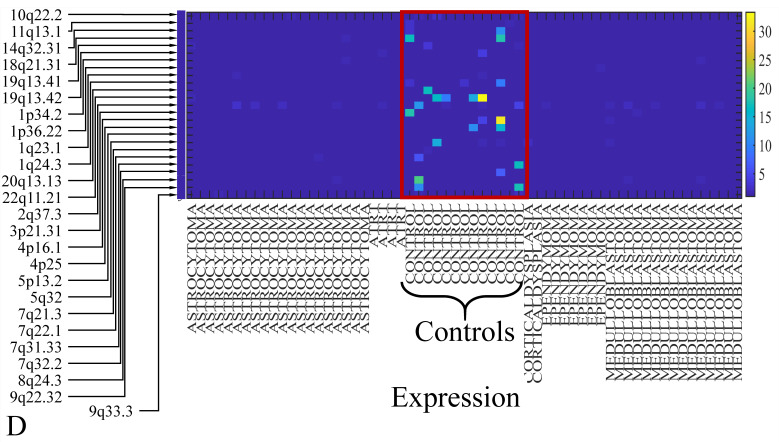

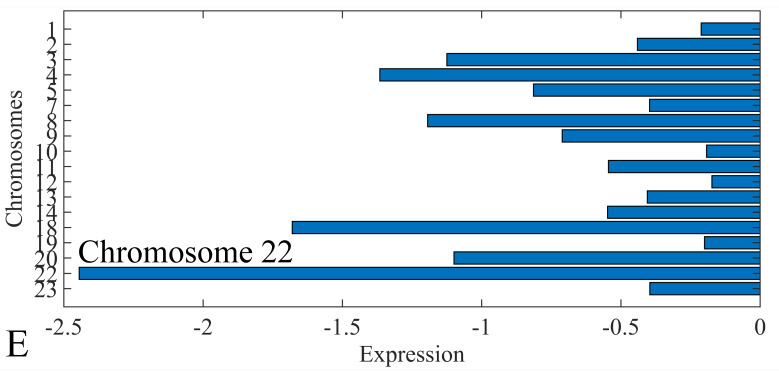

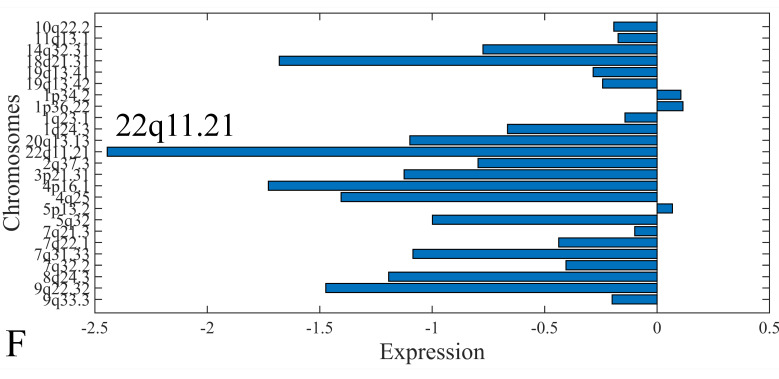

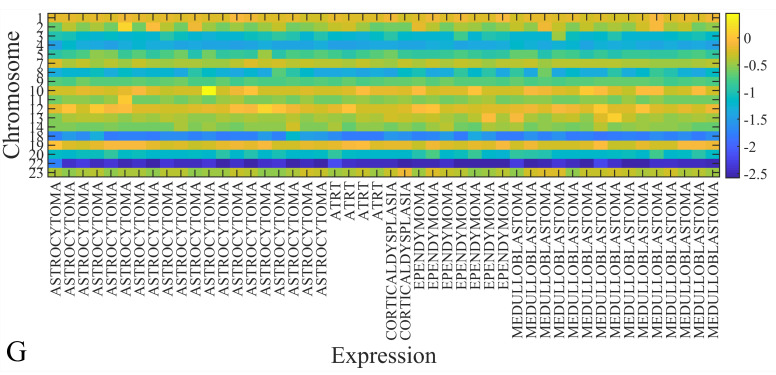

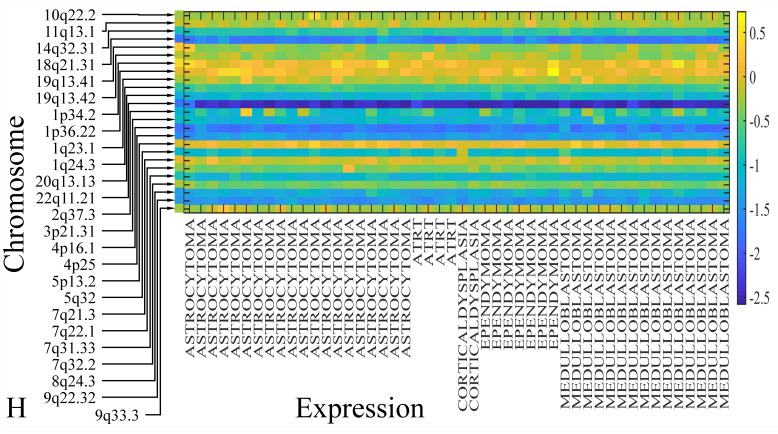
Chromosomal distribution of miRNA expression. miRNA expression, including control samples (“naturals”) was mostly active on chromosome 22 (**A**) and in particular, on the 22q11.21 location (**B**). When investigating the participation of samples, we have found that control samples contributed mostly to the miRNA expression on chromosome 22 (**C**) and location 22q11.21 (**D**). When calculating the miRNA ratios, chromosome 22 (**E**) and location 22q11.21 (**F**) were the most active locations. In the case of miRNA expression ratios, it appeared that all tumor samples contributed equally to chromosome 22 (**G**) and 22q11.21 (**H**).

The first step in gene expression investigation, was the estimation of chromosomal distributions. In particular, we have investigated miRNA expression using the “natural” values, as well as mRNA ratios. It appeared that the most active chromosome was chromosome 22 (Figure 10A) and, in particular, location 22q11.21 (Figure 10B). It appeared also that the highest expression was mostly attributed to control samples (Figure 10C,D). When accounting for miRNA ratios, chromosome 22 (Figure 10E) and location 22q11.21 (Figure 10F) were still the most active chromosome and location, respectively. Interestingly, miRNA expression appeared to be relatively equally distributed across all chromosomes (Figure 10G,H).

### 3.7. K-Means Clustering

**Figure 11:**
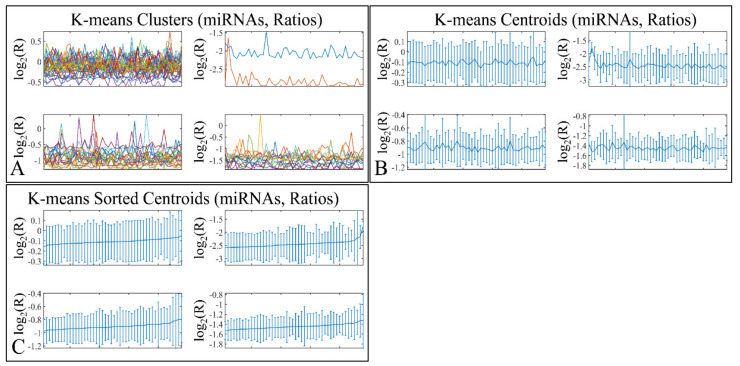
K-means clustering of DE miRNAs. K-means clustering manifested four clusters (**A**), which is presented with the respective centroids (**B**), and sorted centroids (**C**). K-means clusters did not manifest any distinct patterns of expression.

K-means clustering manifested four clusters (Figure 11A), which is presented with the respective centroids (Figure 11B) and sorted centroids (Figure 11C). K-means clusters did not manifest any distinct patterns of expression, as well as sorted centroids did not manifest a certain pattern of expression.

### 3.8. Functional Annotations of DE miRNAs

#### Gene Ontology of DE miRNAs

**Figure 12:**
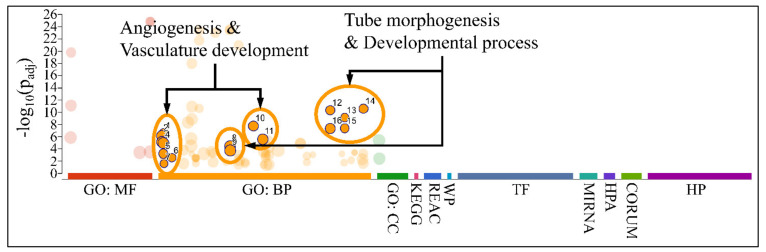
Gene ontology annotation of DE miRNAs. Major functions revealed, included angiogenesis, vasculature development, and developmental processes, such as tube morphogenesis and development (Legend: MF: Molecular function; BP: Biological process; CC: Cellular component; KEGG: KEGG pathway database; REAC: Reactome pathway database; WP: WikiPathways; TF: Transcription factor binding motifs; MIRNA: miRNA targets; HPA: The human protein atlas; CORUM: The comprehensive resource of mammalian protein complexes; HP: Human phenotype ontology).

Functional annotation included gene ontology (GO) annotation of DE miRNAs, which manifested major functions, such as angiogenesis, vasculature development, and developmental processes, such as tube morphogenesis and development (Figure 12). No significant pathways were found for the annotated miRNAs and their gene targets.

### 3.9. Descriptive K-Means

**Figure 13:**
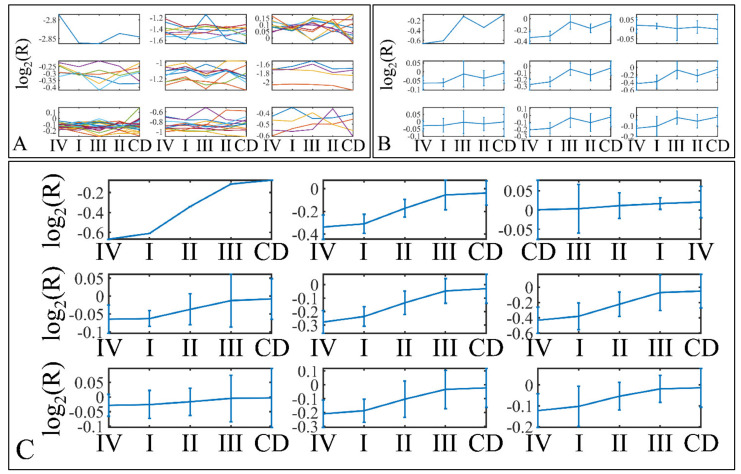
Descriptive k-means of tumor staging (tumor grade). Tumor samples were clustered with respect to the clinical (categorical variables) parameters of tumor grade (**A**,**B**). The first representation concerns the k-means classification of the miRNA expression data (**A**), whereas it is followed by the k-means centroids (**B**). The centroids in (**B**) represent the mean values of the k-means clusters depicted in (**A**). Those centroids (depicted in (**B**)) were sorted in an ascending order, in an attempt to find patterns related to tumor staging (**C**). Indeed, an interesting pattern was revealed with respect to tumor grade, where k-mean sorted centroids (**C**), showed a descending pattern from grade I to III, as well as an ascending pattern from grade III to I (**C**) (Legend: I: grade I (*n* = 16); II: grade II (*n* = 9); III: grade III (*n* = 3); IV: grade IV (*n* = 18); CD: Cortical dysplasia (*n* = 2)).

Descriptive k-means were described in the Section 2. In search of a specific motif or pattern, we have clustered miRNAs with respect to tumor grade (Figure 13A,B) and we have observed that DE miRNAs manifested in all cases a specific pattern, i.e., grade IV tumors manifested lower levels of expression, followed by and ascending order from grade I to III and cortical dysplasia manifesting the highest expression values (Figure 13C). This observation involves probably a trigger mechanism, where DE miRNAs express to similar levels with control samples in near-control tissues (such as cortical dysplasias) and if down-regulated they are connected to aggressive tumor types (IV). The “trigger” mechanism arises probably from the fact that tumor grades I to III manifest an ascending pattern, indicating that those miRNAs operate with certain thresholds.

**Figure 14:**
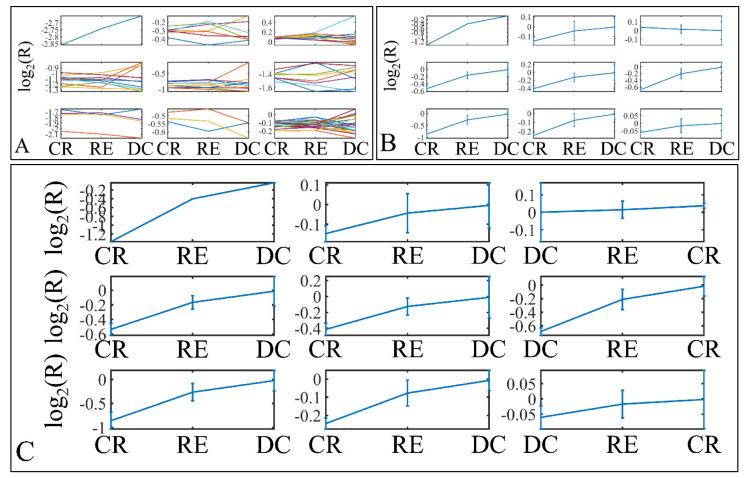
Descriptive k-means of patient survival. Tumor samples were clustered with respect to the patients’ survival profile (**A**,**B**). The first representation concerns the k-means classification of the miRNA expression data (**A**), whereas it is followed by the k-means centroids (**B**). The centroids in (**B**) represent the mean values of the k-means clusters depicted in (**A**). Those centroids (depicted in (**B**)) were sorted in an ascending order, in an attempt to find patterns related to patient survival (**C**). Similarly to tumor staging, an ascending miRNA expression pattern from clinical remission to deceased patients was observed (**C**) (Legend: CR: Clinical remission (*n* = 36); RE: Relapse (*n* = 12); DC: Deceased (*n* = 1)).

Further on, we have also performed k-means clustering with respect to patient survival (Figure 14A,B). Interestingly, all DE miRNAs manifested an ascending pattern when moving from patients who achieved clinical remission, to relapse and deceased patients (Figure 14C). This result indicated that the DE miRNA’s expression levels followed a motif depending on the patient’s survival status.

### 3.10. ROC Analysis

**Figure 15:**
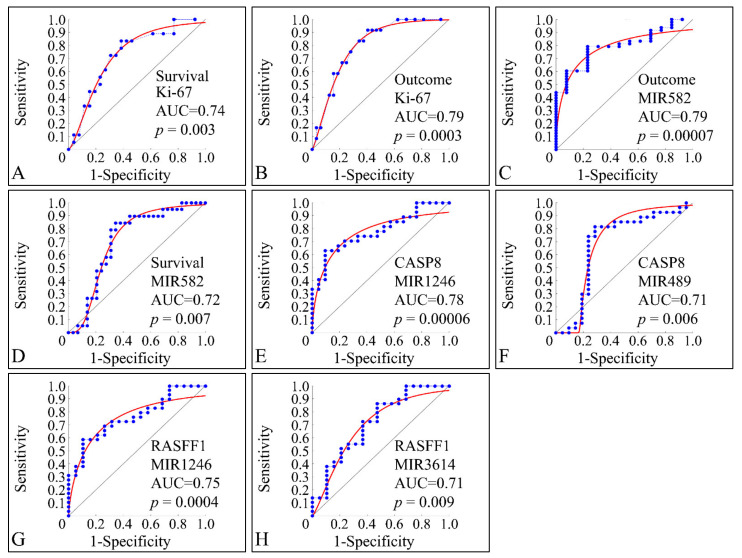
ROC analysis. ROC analysis manifested significant classifiers with respect to clinical parameters. In particular, as expected Ki-67 expression, appeared to classify significantly between alive and deceased patients (described here as survival) (**A**), while it was also able to significantly classify between relapse and clinical remission (described here as outcome) (**B**). Further on, miR-582 appeared to discriminate between clinical remission and relapse (**C**), as well as between alive and deceased patients (**D**). In addition, CASP8 methylation status (i.e., if the gene is methylated or not), was significantly classified by miR-489, while in the case of RASFF1 methylation miR-1246 (**G**) and miR-3614 (**H**) were able to classify between methylated and un-methylated RASFF1.

ROC classification manifested several factors that were able to discriminate between clinical parameters. This type of analysis is able to manifest significant correlations between two variables with respect to a classifier. Only significant classified variables are presented. This type of analysis, basically can indicate that a variable can be distinguished between the parameters of a classifier. As anticipated, Ki-67 expression manifested significant classification potential by discriminating between alive and deceased patients (survival) (Figure 15A), as well as between relapsed patients and patients in clinical remission (outcome) (Figure 15B). Interestingly, miR-582 was also able to classify between outcome (Figure 15C) and survival (Figure 15D). Similarly, miRNA expression was able to discriminate the methylation status of specific genes and in particular miR-1246 (Figure 15E) and miR-489 (Figure 15F) were able to classify this status for CASP8, as well as miR-1246 (Figure 15G) and miR-3614 (Figure 15H) were able to classify for the methylation status of RASFF1.

### 3.11. Naïve-Bayes Analysis

**Figure 16:**
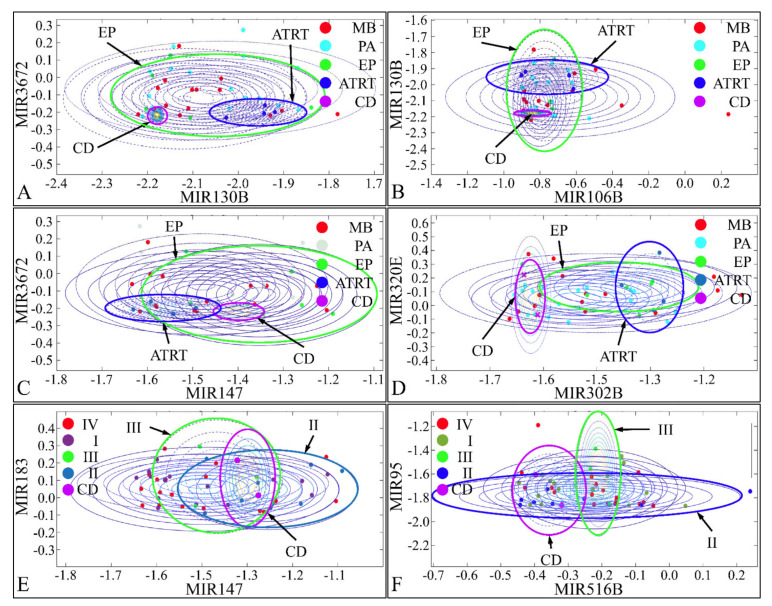
Naïve-Bayes classification. Pairs of miRNAs were tested for their classification potential using naïve-Bayes classifiers. miR-130b and miR-3672 (**A**), miR-106b and miR-130b (**B**), and miR-147 and miR-3672 (**C**) were able to discriminate between CD, ATRT, and EP. Similarly, miR-302b and miR-320e (**D**), miR-147 and miR-183 (**E**), and miR-516b and miR-95 (**F**) were able to discriminate between CD, tumor grade II, and tumor grade III (Legend: CD: Cortical dysplasia; EP: Ependymoma; CD: Cortical dysplasia; PA: Pilocytic astrocytoma; MB: Medulloblastoma; ATRT: Atypical teratoid rhabdoid tumor).

Pairwise clustering of DE miRNAs was examined with a naïve-Bayes classification. Pairs of DE miRNAs were tested for their classification potential and we found that miR-130b and miR-3672 (Figure 16A), miR-106b and miR-130b (Figure 16B), and miR-147 and miR-3672 (Figure 16C) were able to discriminate between CD, ATRT, and EP. Similarly, miR-302b and miR-320e (Figure 16D), miR-147 and miR-183 (Figure 16E), and miR-516b and miR-95 (Figure 16F) were able to discriminate between CD, tumor grade II, and tumor grade III. Although, we have tested all possible combinations of DE miRNAs for all clinical parameters, only few classifying miRNAs were obtained. Interestingly, the identified miRNAs, could separate ATRT, CD, and EP, while MB and PA manifested similar clusters, i.e., close expression values.

### 3.12. Common miRNA Signatures

**Figure 17:**
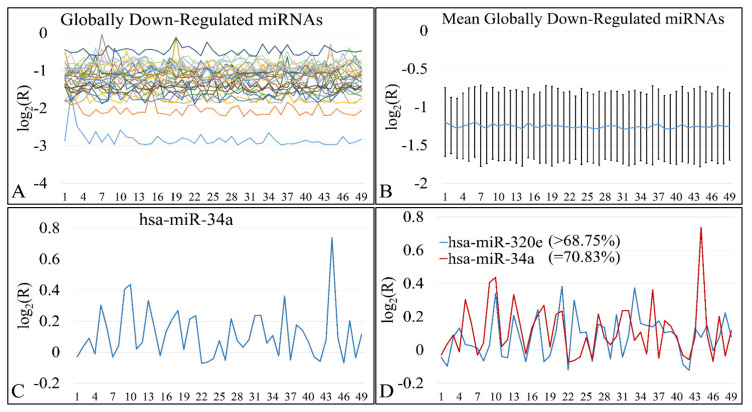
Globally down- and up-regulated miRNAs. 31 miRNAs were found to be globally down-regulated in all tumor samples (**A**,**B**). On the other hand, one miRNA (miR-34a) was found to be globally up-regulated in ~70% of all tumors (**C**), while miR-320e was the next miRNA with ~68% up-regulation in all tumors (**D**).

DE miRNAs manifested a common pattern of expression. In particular, 31 miRNAs were found to be globally down-regulated in all Timor samples (Figure 17A,B). miRNAs included MIR649, MIR130B, MIR4330, MIR95, MIR1226, MIR3123, MIR582, MIR23B, MIR147, MIR302B, MIR214, MIR3942, MIR656, MIR3616, MIR1234, MIR1226, MIR4329, MIR645, MIR592, MIR3202, MIR1246, MIR584, MIR576, MIR4267, MIR4317, MIR542, MIR96, MIR3618, MIR4270, MIR4251, and MIR4307. All miRNAs were down-regulated in all tumor samples without exception (100%). On the other hand, up-regulated miRNAs did not manifest total over-expression in all tumor samples, yet MIR34A appeared to be up-regulated in the 70.83% of tumor samples (Figure 17C). MIR34A up-regulation was followed by MIR320E with over-expression in 68.75% of all tumor samples (Figure 17D). MIR34A is estimated to have approximately 900 mRNA targets. Out of these, 17 genes were differentially expressed in our patient cohort. In particular, these genes were HYAL3, KIAA1210, MYO1C, FAM162B, OPN4, TP53INP2, ZNF281, TTC19, CRHR1, RAD9B, PAX8, JMJD1C, PPP1R11, PDGFRA, SCNN1G, SHKBP1, and ELMOD1. Similarly, MIR320E has 833 predicted mRNA targets, whereas six are differentially expressed in our patient cohort. More specifically, those genes included POLR1C, SIAH3, PLEKHA4, PTP4A1, TLL1, and RCN2. We have further searched for functional annotations of those genes, but no significant results were obtained, probably due to the fact that the gene sample size was small.

## 4. Discussion

In the present study, we have performed high throughput experimentation, which included miRNA microarrays, mRNA microarrays and methylation studies with MS-MLPA. Our basic aim was to detect both differential, as well as common miRNA expression patterns and connect them to the expressed mRNA patterns and in addition, find their relations to specific gene methylation. Our analysis, revealed a total of 75 differentially expressed miRNAs between all CNS tumors and the control cohort. Amongst them, 31 were globally down-regulated and two were globally up-regulated in >68% of all tumors. More specifically, miR-34a and miR-320e were found up-regulated in ~68% of all tumors when compared to the control group. The present observation was in agreement with our previous report on embryonal tumors, where we also found that miR-34a is probably globally up-regulated in tumors. MiR-34a is considered a tumor suppressor gene that it is known to regulate SIRT1 expression (silent information regulator 1). SIRT1, is known to be an oncogene, since it is a key-regulator of tumor suppressor proteins, such as the transcription factor p53 [66,67,68]. Consequently, one might have expected that the elevated expression levels of miR-34a observed in our study in both tumor types when compared with the control group would result in suppression of the SIRT1 expression, leading to apoptosis of cancer cells. A potential response for this came from Yamacuchi et al. (2008), who previously proposed that overexpression of miR-34a does not entirely suppress SIRT1 translation, possibly because it does not exactly match its SIRT1 binding site [67]. MiR-34a overexpression has also been observed in childhood ependymomas [69], pilocytic astrocytomas [70] and in low- and high-grade astrocytomas of childhood [71], suggesting potential global oncogenic roles in pediatric brain malignancies. Similarly, miR-34a regulates PDGFRA [72,73,74] and PAX8 [75], two genes which are known to be involved in childhood CNS tumors, as especially PDGFRA has been studied for its role in gliomas [72,73,74]. In general, there are still very few reports on the connection between miR-34a and those two genes. This indicates that there is a large field of research still open in order to comprehend CNS tumor biology. Yet, another interesting gene-target of miR-34a is the TP53 gene. Although there are numerous reports on the role and correlation between those two molecules, there is only one report concerning its role in the CNS. In particular, it is reported that TP53 is a direct target of miR-34a participating in microglia behavior and suppression of neuro-inflammation [76]. Hence, miR-320e and miR-34a might afford potential biomarkers related to inferior prognosis or even suggest possible global therapeutic targets.

Noteworthy, miR-34a overexpression observed in the present study is in agreement with previous studies, such as the study of Costa et al. (2011) [69], who reported that miR-34a was found to be highly overexpressed in ependymomas, however, in this study it was proposed that miR-34a is linked to a more favorable prognosis. In addition, we have found that miR-320e was significantly up-regulated in our patient population, highlighting a potential for this gene. According to our findings it can be not only as a possible diagnostic biomarker, but also a prognostic factor of a more favorable clinical outcome.

Through our analysis, using a ROC classifier, we have found a set of miRNAs that could pose potential biomarkers for tumor outcome, i.e., clinical remission or relapse. Interestingly, miR-582 was found to be differentially expressed between tumor samples and controls and it appeared to be able to separate samples with respect to survival and therapeutic outcome. The identification of miR-582 is described for the first time for childhood CNS tumors, since there are no previous reports concerning that miRNA. Moreover, we observed that miR-1246, miR-489, and miR-3614 could discriminate between the methylation status of CASP8 and RASFF1.

In the same context, miR-649, miR-130B, miR-4330, miR-95, miR-1226, miR-3123, miR-582, miR-23b, miR-147, miR-302b, miR-214, miR-3942, miR-656, miR-3616, miR-1234, miR-1226, miR-4329, miR-645, mIR-592, miR-3202, miR-1246, miR-584, miR-576, miR-4267, miR-4317, miR-542, miR-96, miR-3618, miR-4270, miR-4251, miR-4307, miR-720, miR-891a, miR-522, miR-518c, miR-3665, miR-3620, miR-382, miR-452, and miR-122 and miR-147 were found to be down-regulated in the patient group compared to control samples, thus indicating that they might participate in the tumor machinery as tumor-suppressor genes. There are some previous studies, reporting a potential link between the aforementioned tumor-suppressor miRNAs with different types of malignancies. First of all, for miR-649, miR-130B, miR-4330, miR-95, miR-1226, miR-3123, miR-147, miR-3942, miR-3616, miR-1234, miR-1226, miR-4329, miR-645, miR-3202, miR-4267, miR-4317, miR-3618, miR-4251, miR-4307, miR-720, miR-891a, miR-518c, miR-3665, miR-3620, and miR-147 there are no previous reports for their role in childhood CNS tumors but also in CNS tumors in general and, therefore, this is the first time they are referred as playing a role in CNS tumor pathophysiology. On the other hand, reports on the remaining miRNAs have been controversial for some. For example, miR-34a is reported to act as tumor suppressor miRNA, while we have identified it as a possible oncogene. Further on, our study is in agreement with respect to the role of miR-320e, where a previous report has highlighted that it manifests oncogenic properties [77]. In order to obtain a more “panoramic” view of the identified miRNAs we have summarized our common de-regulated miRNAs along with the reported functions in the literature (Table 5).

Interestingly, out of the globally deregulated miRNAs, miR-3202, miR-4251, and miR-4270, were found to be significantly different with respect to gender. To the best of our knowledge, there are no reports concerning the role of miRNAs in gender-specific CNS tumor ontogenesis and progression. Yet, few reports, which drew our attention, indicated that there is a gender-specific tumorigenesis for gliomas. In particular, it has been reported that females had a predominance in developing CNS tumors in case of previous cancer familial history, indicating a possible hereditary gender-specific risk [142]. Another interesting perspective of gender-specific visualization, has to do with the possible personalized treatments for patients. In particular, it has been reported that the adenine-to-inosine “inosinome” [143], is a potent gender-specific glioblastoma stratifier [144]. Although, there are no previous reports on the role of miRNAs with respect to gender, this approach could prove useful in towards a personalized treatment for CNS tumors.

Ki-67 protein is present during all active phases of the cell cycle making it an excellent marker to predict cell proliferation [145]. In the current setting we investigated the potential interactions between Ki-67 ‘positive’ and miRNA expression patterns, in order to unravel and characterize the role of miRNAs underlying pediatric embryonal brain tumors. All potential miRNA oncogenes and tumor-suppressive genes that emerged from previous correlations between miRNA expression profiles and disease progression or patient clinical outcome were yet again further demonstrated as elevated and decreased expression levels were observed in the Ki-67 ‘positive’ group of patients versus control tissues, respectively. In addition, identical findings were manifested regarding their putative prognostic role, either favorable or inferior. Yet, again, our prediction regarding their prognostic significance in pediatric embryonal CNS neoplasms was confirmed.

In the present work, we have also attempted to identify methylation and CNV patterns in childhood CNS tumors. Since the first reports on the role of DNA methylation and its significance in epigenetic regulation, numerous reports have highlighted its role in cancer. In particular, several reports have outlined its significance in pediatric CNS tumors [146]. In the present work, we have found that methylation played a role with respect to its number of methylated genes and not with respect to the individual methylated genes. This finding, to the best of our knowledge, is reported for the first time and it implies that epigenetic regulation is significant in tumor progression and it is probably the result of a multifactorial process.

Previous reports have indicated that supratentorial primitive neuroectodermal tumors (PNET), as well as ATRT manifest an aberrant RASSF1A methylation but not CASP8 [147]. In the present work, in a single ATRT sample, we have found methylation in the MGMT gene. This finding was different from a previous retrospective observational study, where it was found that most ATRT cases did not manifested a MGMT methylation [148].

In our work, we have found that five MBs manifested a CASP8 methylation while two did not manifest any methylation. Our finding was in agreement with previous studies, where CASP8 was also found to be methylated indicating a significant role of CASP8 in MB pathophysiology [149,150]. Epigenetic regulation of CASP8 signifies the role of pro-apoptotic molecules in tumor progression. At the same time six samples were found to be methylated in the RASSF1 gene, which is in agreement with previous studies which indicated the significance of RASSF1 in MB pathology [151,152,153]. It appears that RASSF1 methylation leads to RASSF1 deactivation indicating a significant role of RASSF1 to MB pathology [151].

In the case of PA tumors, most samples appeared to be unmethylated and only three samples were found to be methylated in the CASP8, RASSF1, and MGMT genes. To the best of our knowledge, there are no previous reports on gene methylation of pediatric PA. This result was confirmed with the finding of our study that methylation status was significantly related to tumor grade.

Similarly, the two EP tumors we investigated were found to possess a methylation on MSH6 and RASSF1 genes. There are no previous reports concerning the methylation status of pediatric EP tumors.

Interestingly, we have found that several miRNAs including miR-128, miR-183, miR-3202, miR-302e, miR-4307, miR-4330, and miR-491-3p manifested significant differences between the methylation status of CASP8 and RASSF1. Although these miRNAs were not found to consist of a target for CASP8 and RASSF1, it was interesting to observe that those differences were manifested irrespectively of tumor type. To the best of our knowledge there are no previous reports on the correlation of those miRNAs to the methylation status of CASP8 and RASSF1 in CNS tumors.

Our understanding of the exact miRNA mechanisms in childhood CNS tumors’ machinery is still limited. Thus, the comprehension of these mechanisms, regarding CNS tumor pathogenesis, could reveal candidate therapeutic targets. Further on, miRNAs could be used as possible biomarkers, for the prognosis, diagnosis, and treatment of childhood CNS tumors, but their role still remains largely unexplored.

MicroRNAs offer insights to many processes of the human body and are fascinating molecules to investigate. However, the study of miRNAs often involves hybridization-based microarray technologies, a high-throughput technology, that may generate a large opportunity for errors when used to test miRNAs. Due to these limitations, all experiments must be regulated and controlled, to reduce the chances of error regarding the data produced. In addition, other technologies must be implemented as a way to confirm the results of the microarrays. A commonly used technique is RT-PCR, which amplifies specific genes as a way to validate the microarrays.

## 5. Conclusions

In summary, the present study attempted to provide insight into the growing role of several miRNA signatures in pediatric CNS neoplasms. We have found that miR-34a and miR-320e were globally up-regulated in the majority of brain tumors, as well as miR-649, miR-130B, miR-4330, miR-95, miR-1226, miR-3123, miR-582, miR-23b, miR-147, miR-302b, miR-214, miR-3942, miR-656, miR-3616, miR-1234, miR-1226, mIR-4329, miR-645, mIR-592, miR-3202, miR-1246, miR-584, miR-576, miR-4267, miR-4317, miR-542, miR-96, miR-3618, miR-4270, miR-4251, miR-4307, miR-720, miR-891a, miR-522, miR-518c, miR-3665, miR-3620, miR-382, miR-452 and miR-122, and miR-147 were globally down-regulated in childhood brain tumors. Generally, there was good evidence that the aforementioned miRNA signatures could serve as: (a) Oncogenic diagnostic molecules; (b) Indicators of favorable prognosis when overexpressed; and inferior prognosis when up-regulated. Overall, our findings suggested novel molecular biomarkers which might have a promising potential in pediatric embryonal CNS malignancies. In the present work, we have also investigated the methylation status of pediatric CNS tumors. We have found that CASP8, RASSF1 were the most frequently methylated. Finally, MSH6 was found methylated in one EP sample. Epigenetic regulation appears to be of major importance in tumor progression and pathophysiology, making it an imperative field of study.

## Figures and Tables

**Table 1:** Demographic and clinical characteristics of the study population (Legend: StDev: Standard deviation, ATRT: Atypical teratoid rhabdoid tumor, CR: Clinical remission).

Total Population (*n* = 62)								
	Mean ± StDev	Median	Min			Max		
Age at Diagnosis (years)	6.20 ± 4.33	6.20	0.03			16.06		
Time of Curation	1051.05 ± 5295.79	248.75	175.00			38,112.00		
Survival (years)	8.48 ± 3.65	7.93	0.78			17.98		
**With Respect to Gender**								
			Males (*n* = 39)			Females (*n* = 25)		
	Mean ± StDev	Median	Min	Max	Mean ± StDev	Median	Min	Max
Age at Diagnosis (years)	6.59 ± 4.59	4.92	0.03	16.06	5.72 ± 4.04	6.69	0.26	13.52
Time of Curation (Days)	321.64 ± 188.57	248.75	175.00	1051.05	1871.63 ± 7720.16	246.50	177.17	38,112.00
Survival (years)	8.49 ± 3.38	7.94	1.39	17.98	8.46 ± 4.13	7.70	0.78	17.93
**With Respect to Diagnosis**								
	ASTROCYTOMA (*n* = 20) Mean ± StDev Median (Min-Max)	EPENDYMOMA (*n* = 7) Mean ± StDev Median (Min-Max)	MEDULLOBLASTOMA (*n* = 16) Mean ± StDev Median (Min-Max)		ATRT (*n* = 4) Mean ± StDev Median (Min-Max)	CORTICAL DYSPLASIA (*n* = 2) Mean ± StDev Median (Min-Max)		CONTROL (*n* = 13) Mean ± StDev Median (Min-Max)
Age at Diagnosis (years)	5.86 ± 3.42 3.42 (0.92–13.05)	5.13 ± 5.40 5.40 (1.44–16.01)	6.92 ± 4.70 4.70 (0.74–16.06)		2.46 ± 3.54 3.54 (0.03–7.61)	10.77 ± 3.87 10.77 (8.04, 13.51)		6.20 ± 0.00 0.00 (6.20–6.20)
Time of Curation (Days)	2263.75 ± 8681.67 8681.67 (177.17–38,112.00)	289.54 ± 133.32 133.32 (175.00–542.17)	342.54 ± 143.61 143.61 (201.67–553.42)		292.25 ± 121.79 121.79 (200.67–471.58)	240.79 ± 19.97 240.79 (226.66, 254.91)		1051.05 ± 0.00 0.00 (1051.05–1051.05)
Survival (years)	8.69 ± 2.74 2.74 (6.22–17.75)	8.28 ± 2.72 2.72 (5.75–12.86)	9.99 ± 5.74 5.74 (1.39–17.98)		5.10 ± 3.80 3.80 (0.78–7.93)	7.91 ± 0.65 7.91 (7.45, 8.38)		8.48 ± 0.00 0.00 (8.48–8.48)
**With Respect to Tumor Grade**								
	Grade I (*n* = 16) Mean ± StDev Median (Min-Max)	Grade II (*n* = 9) Mean ± StDev Median (Min-Max)	Grade III (*n* = 3) Mean ± StDev Median (Min-Max)			Grade IV (*n* = 18) Mean ± StDev Median (Min-Max)		CONTROL (*n* = 15) Mean ± StDev Median (Min-Max)
Age at Diagnosis (years)	6.47 ± 3.37 6.62 (1.99–13.05)	5.94 ± 5.31 4.47 (0.26–16.01)	1.75 ± 0.33 1.72 (1.44–2.10)			6.33 ± 4.70 7.06 (0.03–16.06)		9.25 ± 3.80 8.04 (6.20–13.52)
Time of Curation (Days)	276.98 ± 111.93 241.46 (189.17–553.42)	4041.37 ± 11,971.66 238.96 (177.17–38,112.00)	281.33 ± 108.08 277.92 (175.00–391.08)			337.28 ± 140.07 254.42 (200.67–553.42)		501.93 ± 475.76 241.50 (213.25–1051.05)
Survival (years)	8.58 ± 2.79 7.94 (6.22–17.75)	7.75 ± 1.31 7.93 (6.25–9.99)	9.31 ± 5.02 9.31 (5.75–12.86)			8.84 ± 5.88 7.04 (0.78–17.98)		7.81 ± 0.74 7.94 (7.01–8.48)
**With Respect to Clinical Outcome**								
	CR (*n* = 36) Mean ± StDev Median (Min-Max)		RELAPSE (*n* = 12) Mean ± StDev Median (Min-Max)					CONTROL (13) Mean ± StDev Median (Min-Max)
Age at Diagnosis (years)	6.33 ± 4.31 6.62 (0.03–16.01)		6.22 ± 4.81 5.76 (0.74–16.06)					6.20 ± 0.00 6.20 (6.20–6.20)
Time of Curation (Days)	1350.27 ± 6397.47 241.25 (175.00–38,112.00)		386.45 ± 140.85 407.71 (204.42–553.42)					1051.05 ± 0.00 1051.05 (1051.05–1051.05)
Survival (years)	8.60 ± 3.09 7.92 (5.75–17.98)		7.68 ± 7.03 8.33 (0.78–17.93)					8.48 ± 0.00 8.48 (8.48–8.48)

**Table 2:** Primer design for methylated and unmethylated RASSF1 and CASP8 genes.

Primers for MS-PCR for RASSF1 Promoter	
Primer	Sequence
Methylated	
Left RASSF1 F primer	GTAAAGTTGGTTTTTAGAAATACGG
Right RASSF1 R primer	AAAAAAAACTAAAAAAAACCGCG
Product size: 212, Tm: 69.30	
Unmethylated	
Left RASSF1 F primer	GTAAAGTTGGTTTTTAGAAATATGG
Right RASSF1 R primer	AAAAAAAACTAAAAAAAACCACACA
Product size: 212, Tm: 69.30	
**Primers for MS-PCR for CASP8 Promoter**	
Primer	Sequence
Methylated	
Left CASP8 F primer	TGGGAGTAAGGTAGAGTTAGAGGTC
Right CASP8 R primer	AATTTCAAATCCCAAATTATTTCG
Product size: 142, Tm: 66.90	
Unmethylated	
Left CASP8 F primer	GGGAGTAAGGTAGAGTTAGAGGTTG
Right CASP8 R primer	AAATTTCAAATCCCAAATTATTTCA
Product size: 142, Tm: 66.90	

**Table 3:** Relations of methylated genes with respect to sampling. The data represents the number (*n*) of samples found to be methylated in each subcategory (genes separated with ‘/’ imply the simultaneous methylation of all genes). In particular, we have investigated the relation of methylation with respect to diagnosis and tumor grading. The table depicts the number of samples found to have their promoter methylated with respect to their diagnosis, i.e., between (a) neoplasms and controls; (b) malignant, benign, and control tissues; (c) medulloblastoma, astrocytoma, ATRT, ependymoma, and controls; and (d) tumor staging.

Diagnosis vs. Methylated Genes	NONE	CASP8/RASSF1 (*n*)	MGMT (*n*)	CASP8/RASSF1/CD44 (*n*)	CASP8 (*n*)	MSH6 (*n*)	CASP8/GATA5/MGMT/ATM1/TP53 (*n*)	CASP8/RASSF1/CD44/CADM1/RB1 (*n*)	RASS1 (*n*)	SUM
NEOPLASMS (*n*)	15	12	2	1	1	1	0	1	2	35
CONTROLS (*n*)	7	0	0	0	3	0	1	0	0	11
SUM	22	12	2	1	4	1	1	1	2	46
Chi-square	16.19									
*p*-value	0.040									
Diagnosis vs. Methylated Genes	**NONE**	**CASP8/RASSF1**	**MGMT**	**CASP8/RASSF1/CD44**	**CASP8**	**MSH6**	**CASP8/GATA5/MGMT/ATM1/TP53**	**CASP8/RASSF1/CD44/CADM1/RB1**	**RASSF1**	**SUM**
MALIGNANCY	4	8	1	1	0	1	0	1	2	18
BENIGN	11	4	1	0	1	0	0	0	0	17
CONTROL	7	1	0	0	3	0	1	0	0	12
SUM	22	13	2	1	4	1	1	1	2	47
Chi-square	30.90									
*p*-value	0.014									
Diagnosis vs. Methylated Genes	**NONE**	**CASP8/RASSF1**	**MGMT**	**CASP8/RASSF1/CD44**	**CASP8**	**MSH6**	**CASP8/GATA5/MGMT/ATM1/TP53**	**CASP8/RASSF1/CD44/CADM1/RB1**	**RASSF1**	**SUM**
MEDULLOBLASTOMA	4	8	0	1	0	0	0	1	1	15
ASTROCYTOMA	11	4	1	0	1	0	0	0	0	17
ATRT	0	0	1	0	0	0	0	0	0	1
CONTROL	7	1	0	0	3	0	1	0	0	12
EPENDYMOMA	0	0	0	0	0	1	0	0	1	2
SUM	22	13	2	1	4	1	1	1	2	47
Chi-square	65.86									
*p*-value	0.0004									
Grade vs. Methylated Genes	**NONE**	**CASP8/RASSF1**	**MGMT**	**CASP8/RASSF1/CD44**	**CASP8**	**MSH6**	**CASP8/GATA5/MGMT/ATM1/TP53**	**CASP8/RASSF1/CD44/CADM1/RB1**	**RASSF1**	**SUM**
IV	4	7	1	1	0	0	0	1	1	15
I	9	1	0	0	1	0	0	0	0	11
II	2	3	1	0	0	1	0	0	0	7
CONTROL	7	1	0	0	3	0	1	0	0	12
III	0	0	0	0	0	0	0	0	1	1
SUM	22	12	2	1	4	1	1	1	2	46
Chi-square	55.55									
*p*-value	0.0061									

**Table 4:** Relations of copy number variations (CNV) with respect to sampling. The data represent the number (*n*) of samples found with CNV in each subcategory (CNVs separated with ‘/’ imply the simultaneous presence of those). In particular, we have investigated the relation of gene aberrations with respect to diagnosis and tumor grading. In particular, we have investigated the relation of gene aberrations with respect to diagnosis and tumor grading. The table depicts the number of samples found to manifest a gene aberration with respect to their diagnosis, i.e., between (a) neoplasms and controls; (b) malignant, benign, and control tissues; (c) medulloblastoma, astrocytoma, ATRT, ependymoma, and controls; and (d) tumor staging.

Diagnosis vs. Aberrations	NONE	CD27 DUPLICATION (*n*)	CD6 DELETION (*n*)	CD6/CDKN2A/GATA5 DELETION (*n*)	CD44 DUPLICATION (*n*)	SUM
NEOPLASM (*n*)	33	1	1	1	0	36
CONTROL (*n*)	10	0	0	0	1	11
SUM	43	**1**	**1**	**1**	**1**	47
Chi-square	4.18					
*p*-value	0.38					
Diagnosis vs. Aberrations	**NONE**	**CD27 DUPLICATION**	**CD6 DELETION**	**CD6/CDKN2A/GATA5** **DELETION**	**CD44 DUPLICATION**	**SUM**
MALIGNANCY	18	0	0	0	0	18
BENIGN	14	1	1	1	0	17
CONTROL	11	0	0	0	1	12
SUM	43	**1**	**1**	**1**	**1**	47
Chi-square	7.97					
*p*-value	0.44					
Diagnosis vs. Aberrations	**NONE**	**CD27 DUPLICATION**	**CD6 DELETION**	**CD6/CDKN2A/GATA5** **DELETION**	**CD44 DUPLICATION**	**SUM**
MEDULLOBLASTOMA	15	0	0	0	0	15
ASTROCYTOMA	14	1	1	1	0	17
ATRT	1	0	0	0	0	1
CONTROL	11	0	0	0	1	12
EPENDYMOMA	2	0	0	0	0	2
SUM	43	**1**	**1**	**1**	**1**	47
Chi-square	8.51					
*p*-value	0.93					
Diagnosis vs. Aberrations	**NONE**	**CD27 DUPLICATION**	**CD6 DELETION**	**CD6/CDKN2A/GATA5** **DELETION**	**CD44 DUPLICATION**	**SUM**
IV	15	0	0	0	0	15
I	8	1	1	1	0	11
II	7	0	0	0	0	7
CONTROL	11	0	0	0	1	12
III	1	0	0	0	0	1
SUM	42	1	1	1	1	46
Chi-square	12.98					
*p*-value	0.67					

**Table 5:** Comparative table of the miRNAs identified in the present study and those reported in the literature with respect to their expressional profiles and reported functions.

Inv.	miRNA	Expression (Present Study)	Suspected Function (Present Study)	Expression (in the Literature)	Reported Function (in the Literature)	Therapeutic Target?	References
1	miR-34a	Up-regulated	Oncogene	Down-regulated	Tumor suppressor	Yes	[78,79,80,81,82,83,84,85,86,87,88,89,90,91]
2	miR-320e	Up-regulated	Oncogene	Up-regulated	Oncogene	Yes	[77]
3	miR-649	Down-regulated	Tumor suppressor	Not known	Not known	Not known	None available
4	miR-130B	Down-regulated	Tumor suppressor	Not known	Not known	Not known	None available
5	miR-4330	Down-regulated	Tumor suppressor	Not known	Not known	Not known	None available
6	miR-95	Down-regulated	Tumor suppressor	Not known	Not known	Not known	None available
7	miR-1226	Down-regulated	Tumor suppressor	Not known	Not known	Not known	None available
8	miR-3123	Down-regulated	Tumor suppressor	Not known	Not known	Not known	None available
9	miR-147	Down-regulated	Tumor suppressor	Not known	Not known	Not known	None available
10	miR-3942	Down-regulated	Tumor suppressor	Not known	Not known	Not known	None available
11	miR-3616	Down-regulated	Tumor suppressor	Not known	Not known	Not known	None available
12	miR-1234	Down-regulated	Tumor suppressor	Not known	Not known	Not known	None available
13	miR-1226	Down-regulated	Tumor suppressor	Not known	Not known	Not known	None available
14	mIR-4329	Down-regulated	Tumor suppressor	Not known	Not known	Not known	None available
15	miR-645	Down-regulated	Tumor suppressor	Not known	Not known	Not known	None available
16	miR-3202	Down-regulated	Tumor suppressor	Not known	Not known	Not known	None available
17	miR-4267	Down-regulated	Tumor suppressor	Not known	Not known	Not known	None available
18	miR-4317	Down-regulated	Tumor suppressor	Not known	Not known	Not known	None available
19	miR-3618	Down-regulated	Tumor suppressor	Not known	Not known	Not known	None available
20	miR-4251	Down-regulated	Tumor suppressor	Not known	Not known	Not known	None available
21	miR-4307	Down-regulated	Tumor suppressor	Not known	Not known	Not known	None available
22	miR-720	Down-regulated	Tumor suppressor	Not known	Not known	Not known	None available
23	miR-891a	Down-regulated	Tumor suppressor	Not known	Not known	Not known	None available
24	miR-518c	Down-regulated	Tumor suppressor	Not known	Not known	Not known	None available
25	miR-3665	Down-regulated	Tumor suppressor	Not known	Not known	Not known	None available
26	miR-3620	Down-regulated	Tumor suppressor	Not known	Not known	Not known	None available
27	miR-147	Down-regulated	Tumor suppressor	Not known	Not known	Not known	None available
28	miR-582	Down-regulated	Tumor suppressor	Up-regulated ^1^	Oncogene	Not known	[92]
29	miR-23b	Down-regulated	Tumor suppressor	Up-regulated	Oncogene	Not known	[93,94,95,96]
30	miR-23b	Down-regulated	Tumor suppressor	Down-regulated	Tumor suppressor	Not known	[97,98,99]
31	miR-302b	Down-regulated	Tumor suppressor	Down-regulated	Tumor suppressor	Not known	[100]
32	miR-214	Down-regulated	Tumor suppressor	Up-regulated	Oncogene	Not known	[101,102,103,104,105]
33	miR-214	Down-regulated	Tumor suppressor	Down-regulated	Tumor suppressor	Not known	[106,107,108,109,110,111]
34	mIR-656	Down-regulated	Tumor suppressor	Down-regulated	Tumor suppressor	Not known	[106,107,108,109,110,111]
35	miR-592	Down-regulated	Tumor suppressor	Down-regulated	Tumor suppressor	Not known	[95,112,113]
36	miR-1246	Down-regulated	Tumor suppressor	Up-regulated	Oncogene	Not known	[114]
37	miR-1246	Down-regulated	Tumor suppressor	Down-regulated	Tumor suppressor	Not known	[115]
38	miR-584	Down-regulated	Tumor suppressor	Down-regulated	Tumor suppressor	Not known	[116,117,118,119,120,121]
39	miR-576	Down-regulated	Tumor suppressor	Down-regulated	Tumor suppressor	Not known	[122]
40	miR-576	Down-regulated	Tumor suppressor	Up-regulated	Oncogene	Not known	[123]
41	miR-542	Down-regulated	Tumor suppressor	Up-regulated	Oncogene	Yes	[124]
42	miR-96	Down-regulated	Tumor suppressor	Down-regulated	Tumor suppressor	Yes	[125,126,127,128,129,130]
43	miR-4270	Down-regulated	Tumor suppressor	Up-regulated	Oncogene	Not known	[131]
44	miR-522	Down-regulated	Tumor suppressor	Up-regulated	Oncogene	Not known	[132]
45	miR-382	Down-regulated	Tumor suppressor	Down-regulated	Tumor suppressor	Not known	[133,134]
46	miR-452	Down-regulated	Tumor suppressor	Down-regulated	Tumor suppressor	Not known	[127]
47	miR-122	Down-regulated	Tumor suppressor	Down-regulated	Tumor suppressor	Yes	[135,136,137,138,139,140,141]

^1^ miR-582 is reported to be up-regulated in pituitary adenomas, yet reports on other tumors refer to it as down-regulated and as a tumor suppressor.

## Data Availability

Data are available from the corresponding author upon reasonable request.

## Supplementary Materials

The following are available online at https://www.mdpi.com/article/10.3390/cancers13215491/s1, Figure S1: Flow chart of the present work; Figure S2: Indicative analysis results as they are obtained from the special software; Table S1: The complete list of all differentially expressed miRNAs.

## References

[1] Louis D.N., Ohgaki H., Wiestler O.D., Cavenee W.K., Burger P.C., Jouvet A., Scheithauer B.W., Kleihues P. (2007). The 2007 WHO classification of tumours of the central nervous system. Acta Neuropathol..

[2] Louis D.N., Perry A., Reifenberger G., Von Deimling A., Figarella-Branger D., Cavenee W.K., Ohgaki H., Wiestler O.D., Kleihues P., Ellison D.W. (2016). The 2016 World Health Organization classification of tumors of the central nervous system: A summary. Acta Neuropathol..

[3] Braoudaki M., Lambrou G.I., Giannikou K., Milionis V., Stefanaki K., Birks D.K., Prodromou N., Kolialexi A., Kattamis A., Spiliopoulou C.A. (2014). Microrna expression signatures predict patient progression and disease outcome in pediatric embryonal central nervous system neoplasms. J. Hematol. Oncol..

[4] Braoudaki M., Lambrou G.I., Giannikou K., Papadodima S.A., Lykoudi A., Stefanaki K., Sfakianos G., Kolialexi A., Tzortzatou-Stathopoulou F., Tzetis M. (2016). Mir-15a and mir-24-1 as putative prognostic microrna signatures for pediatric pilocytic astrocytomas and ependymomas. Tumour Biol. J. Int. Soc. Oncodev. Biol. Med..

[5] Braoudaki M., Lambrou G.I., Papadodima S.A., Stefanaki K., Prodromou N., Kanavakis E. (2016). Microrna expression profiles in pediatric dysembryoplastic neuroepithelial tumors. Med. Oncol..

[6] Takei H., Bhattacharjee M.B., Rivera A., Dancer Y., Powell S.Z. (2007). New immunohistochemical markers in the evaluation of central nervous system tumors: A review of 7 selected adult and pediatric brain tumors. Arch. Pathol. Lab. Med..

[7] Suri V.S., Tatke M., Singh D., Sharma A. (2004). Histological spectrum of ependymomas and correlation of p53 and ki-67 expression with ependymoma grade and subtype. Indian J. Cancer.

[8] Scholzen T., Gerdes J. (2000). The ki-67 protein: From the known and the unknown. J. Cell. Physiol..

[9] McDonald M.W., Wolanski M.R., Simmons J.W., Buchsbaum J.C. (2013). Technique for sparing previously irradiated critical normal structures in salvage proton craniospinal irradiation. Radiat. Oncol..

[10] Cohen A.L., Piccolo S.R., Cheng L., Soldi R., Han B., Johnson W.E., Bild A.H. (2013). Genomic pathway analysis reveals that ezh2 and hdac4 represent mutually exclusive epigenetic pathways across human cancers. BMC Med. Genom..

[11] Glass C., Wilson M., Gonzalez R., Zhang Y., Perkins A.S. (2014). The role of evi1 in myeloid malignancies. Blood Cells Mol. Dis..

[12] Larmonie N.S.D., Arentsen-Peters T., Obulkasim A., Valerio D., Sonneveld E., Danen-van Oorschot A.A., De Haas V., Reinhardt D., Zimmermann M., Trka J. (2018). Mn1 overexpression is driven by loss of dnmt3b methylation activity in inv(16) pediatric aml. Oncogene.

[13] Mehdipour P., Murphy T., De Carvalho D.D. (2020). The role of DNA-demethylating agents in cancer therapy. Pharmacol. Ther..

[14] Borchiellini M., Ummarino S., Di Ruscio A. (2019). The bright and dark side of DNA methylation: A matter of balance. Cells.

[15] Mishra R., Haldar S., Suchanti S., Bhowmick N.A. (2019). Epigenetic changes in fibroblasts drive cancer metabolism and differentiation. Endocr. Relat. Cancer.

[16] Baylin S.B. (2002). Mechanisms underlying epigenetically mediated gene silencing in cancer. Semin. Cancer Biol..

[17] Ianniello Z., Fatica A. (2018). N6-methyladenosine role in acute myeloid leukaemia. Int. J. Mol. Sci..

[18] Rongrui L., Na H., Zongfang L., Fanpu J., Shiwen J. (2014). Epigenetic mechanism involved in the hbv/hcv-related hepatocellular carcinoma tumorigenesis. Curr. Pharm. Des..

[19] Tur M.K., Daramola A.K., Gattenlohner S., Herling M., Chetty S., Barth S. (2017). Restoration of dap kinase tumor suppressor function: A therapeutic strategy to selectively induce apoptosis in cancer cells using immunokinase fusion proteins. Biomedicines.

[20] Zhang S.Y., Zhang S.W., Fan X.N., Zhang T., Meng J., Huang Y. (2019). Fundmdeep-m6a: Identification and prioritization of functional differential m6a methylation genes. Bioinformatics.

[21] Singh N., Rashid S., Rashid S., Dash N.R., Gupta S., Saraya A. (2020). Clinical significance of promoter methylation status of tumor suppressor genes in circulating DNA of pancreatic cancer patients. J. Cancer Res. Clin. Oncol..

[22] Liu B., Song J., Luan J., Sun X., Bai J., Wang H., Li A., Zhang L., Feng X., Du Z. (2016). Promoter methylation status of tumor suppressor genes and inhibition of expression of DNA methyltransferase 1 in non-small cell lung cancer. Exp. Biol. Med..

[23] Guzmán L., Depix M.S., Salinas A.M., Roldán R., Aguayo F., Silva A., Vinet R. (2012). Analysis of aberrant methylation on promoter sequences of tumor suppressor genes and total DNA in sputum samples: A promising tool for early detection of copd and lung cancer in smokers. Diagn. Pathol..

[24] Ooki A., Yamashita K., Yamaguchi K., Mondal A., Nishimiya H., Watanabe M. (2013). DNA damage-inducible gene, reprimo functions as a tumor suppressor and is suppressed by promoter methylation in gastric cancer. Mol. Cancer Res..

[25] Sturgeon S.R., Balasubramanian R., Schairer C., Muss H.B., Ziegler R.G., Arcaro K.F. (2012). Detection of promoter methylation of tumor suppressor genes in serum DNA of breast cancer cases and benign breast disease controls. Epigenetics.

[26] Aoki K., Natsume A. (2019). Overview of DNA methylation in adult diffuse gliomas. Brain Tumor Pathol..

[27] Teuber-Hanselmann S., Worm K., Macha N., Junker A. (2021). Mgmt-methylation in non-neoplastic diseases of the central nervous system. Int. J. Mol. Sci..

[28] Kessler T., Sahm F., Sadik A., Stichel D., Hertenstein A., Reifenberger G., Zacher A., Sabel M., Tabatabai G., Steinbach J. (2018). Molecular differences in idh wildtype glioblastoma according to mgmt promoter methylation. Neuro-Oncology.

[29] Michalowski M.B., De Fraipont F., Michelland S., Entz-Werle N., Grill J., Pasquier B., Favrot M.C., Plantaz D. (2006). Methylation of rassf1a and trail pathway-related genes is frequent in childhood intracranial ependymomas and benign choroid plexus papilloma. Cancer Genet. Cytogenet..

[30] Sromek M., Rymkiewicz G., Paziewska A., Szafron L.M., Kulecka M., Zajdel M., Kulinczak M., Dabrowska M., Balabas A., Bystydzienski Z. (2021). A set of 17 micrornas common for brain and cerebrospinal fluid differentiates primary central nervous system lymphoma from non-malignant brain tumors. Biomolecules.

[31] Jelski W., Mroczko B. (2021). Molecular and circulating biomarkers of brain tumors. Int. J. Mol. Sci..

[32] Nadaradjane A., Briand J., Bougras-Cartron G., Disdero V., Vallette F.M., Frenel J.S., Cartron P.F. (2018). Mir-370-3p is a therapeutic tool in anti-glioblastoma therapy but is not an intratumoral or cell-free circulating biomarker. Mol. Ther. Nucleic Acids.

[33] Xu S., Wei J., Wang F., Kong L.Y., Ling X.Y., Nduom E., Gabrusiewicz K., Doucette T., Yang Y., Yaghi N.K. (2014). Effect of mir-142-3p on the m2 macrophage and therapeutic efficacy against murine glioblastoma. J. Natl. Cancer Inst..

[34] Chen Y., Li R., Pan M., Shi Z., Yan W., Liu N., You Y., Zhang J., Wang X. (2017). Mir-181b modulates chemosensitivity of glioblastoma multiforme cells to temozolomide by targeting the epidermal growth factor receptor. J. Neuro-Oncol..

[35] Bhaskaran V., Nowicki M.O., Idriss M., Jimenez M.A., Lugli G., Hayes J.L., Mahmoud A.B., Zane R.E., Passaro C., Ligon K.L. (2019). The functional synergism of microrna clustering provides therapeutically relevant epigenetic interference in glioblastoma. Nat. Commun..

[36] Brat D.J., Scheithauer B.W., Fuller G.N., Tihan T. (2007). Newly codified glial neoplasms of the 2007 who classification of tumours of the central nervous system: Angiocentric glioma, pilomyxoid astrocytoma and pituicytoma. Brain Pathol..

[37] Fuller G.N., Scheithauer B.W. (2007). The 2007 revised world health organization (WHO) classification of tumours of the central nervous system: Newly codified entities. Brain Pathol..

[38] Wu W., Dave N., Tseng G.C., Richards T., Xing E.P., Kaminski N. (2005). Comparison of normalization methods for codelink bioarray data. BMC Bioinform..

[39] Shi L., Reid L.H., Jones W.D., Shippy R., Warrington J.A., Baker S.C., Collins P.J., De Longueville F., Kawasaki E.S., Lee K.Y. (2006). The microarray quality control (maqc) project shows inter- and intraplatform reproducibility of gene expression measurements. Nat. Biotechnol..

[40] Diez D., Alvarez R., Dopazo A. (2007). Codelink: An r package for analysis of ge healthcare gene expression bioarrays. Bioinformatics.

[41] Altman N.S., Hua J. (2006). Extending the loop design for two-channel microarray experiments. Genet. Res..

[42] Churchill G.A. (2002). Fundamentals of experimental design for cdna microarrays. Nat. Genet..

[43] Townsend J.P. (2003). Multifactorial experimental design and the transitivity of ratios with spotted DNA microarrays. BMC Genom..

[44] Furlan D., Sahnane N., Mazzoni M., Pastorino R., Carnevali I., Stefanoli M., Ferretti A., Chiaravalli A.M., La Rosa S., Capella C. (2013). Diagnostic utility of ms-mlpa in DNA methylation profiling of adenocarcinomas and neuroendocrine carcinomas of the colon-rectum. Virchows Arch. Int. J. Pathol..

[45] Herman J.G., Graff J.R., Myohanen S., Nelkin B.D., Baylin S.B. (1996). Methylation-specific pcr: A novel pcr assay for methylation status of cpg islands. Proc. Natl. Acad. Sci. USA.

[46] Li L.C., Dahiya R. (2002). Methprimer: Designing primers for methylation pcrs. Bioinformatics.

[47] Zhang D., Zhang M., Wells M.T. (2006). Multiplicative background correction for spotted microarrays to improve reproducibility. Genet. Res..

[48] Cleveland W.S. (1979). Robust locally weighted regression and smoothing scatterplots. J. Am. Stat. Assoc..

[49] Uzman B.G., Foley G.E., Farber S., Lazarus H. (1966). Morphologic variations in human leukemic lymphoblasts (ccrf-cem cells) after long-term culture and exposure to chemotherapeutic agents. A study with the electron microscope. Cancer.

[50] Yang I.V., Chen E., Hasseman J.P., Liang W., Frank B.C., Wang S., Sharov V., Saeed A.I., White J., Li J. (2002). Within the fold: Assessing differential expression measures and reproducibility in microarray assays. Genome Biol..

[51] Klipper-Aurbach Y., Wasserman M., Braunspiegel-Weintrob N., Borstein D., Peleg S., Assa S., Karp M., Benjamini Y., Hochberg Y., Laron Z. (1995). Mathematical formulae for the prediction of the residual beta cell function during the first two years of disease in children and adolescents with insulin-dependent diabetes mellitus. Med. Hypotheses.

[52] Storey J.D., Tibshirani R. (2003). Statistical significance for genomewide studies. Proc. Natl. Acad. Sci. USA.

[53] Storey J.D., Tibshirani R. (2003). Statistical methods for identifying differentially expressed genes in DNA microarrays. Methods Mol. Biol..

[54] Forgy E.W. (1965). Cluster analysis of multivariate data: Efficiency vs. interpretability of classifications, 1965. Biometrics.

[55] Lloyd S. (1982). Least squares quantization in pcm. IEEE Trans. Inf. Theory.

[56] Freyhult E., Landfors M., Onskog J., Hvidsten T.R., Ryden P. (2010). Challenges in microarray class discovery: A comprehensive examination of normalization, gene selection and clustering. BMC Bioinform..

[57] Gibbons F.D., Roth F.P. (2002). Judging the quality of gene expression-based clustering methods using gene annotation. Genome Res..

[58] Lambrou G., Braoudaki M. (2016). A novel method for the analysis of gene expression microarray data with k-means clustering: Sorted k-means. Int. J. Eng. Res. Sci..

[59] Raudvere U., Kolberg L., Kuzmin I., Arak T., Adler P., Peterson H., Vilo J. (2019). G:Profiler: A web server for functional enrichment analysis and conversions of gene lists (2019 update). Nucleic Acids Res..

[60] Zhang B., Schmoyer D., Kirov S., Snoddy J. (2004). Gotree machine (gotm): A web-based platform for interpreting sets of interesting genes using gene ontology hierarchies. BMC Bioinform..

[61] Steinfeld I., Navon R., Ach R., Yakhini Z. (2012). Mirna target enrichment analysis reveals directly active mirnas in health and disease. Nucleic Acids Res..

[62] Eden E., Navon R., Steinfeld I., Lipson D., Yakhini Z. (2009). Gorilla: A tool for discovery and visualization of enriched go terms in ranked gene lists. BMC Bioinform..

[63] Kern F., Fehlmann T., Solomon J., Schwed L., Grammes N., Backes C., Van Keuren-Jensen K., Craig D.W., Meese E., Keller A. (2020). Mieaa 2.0: Integrating multi-species microrna enrichment analysis and workflow management systems. Nucleic Acids Res..

[64] Li J., Han X., Wan Y., Zhang S., Zhao Y., Fan R., Cui Q., Zhou Y. (2018). Tam 2.0: Tool for microrna set analysis. Nucleic Acids Res..

[65] Lu M., Shi B., Wang J., Cao Q., Cui Q. (2010). Tam: A method for enrichment and depletion analysis of a microrna category in a list of micrornas. BMC Bioinform..

[66] Knight J.R., Allison S.J., Milner J. (2013). Active regulator of sirt1 is required for cancer cell survival but not for sirt1 activity. Open Biol..

[67] Yamakuchi M., Ferlito M., Lowenstein C.J. (2008). Mir-34a repression of sirt1 regulates apoptosis. Proc. Natl. Acad. Sci. USA.

[68] Yamakuchi M. (2012). Microrna regulation of sirt1. Front. Physiol..

[69] Costa F.F., Bischof J.M., Vanin E.F., Lulla R.R., Wang M., Sredni S.T., Rajaram V., de Fátima Bonaldo M., Wang D., Goldman S. (2011). Identification of micrornas as potential prognostic markers in ependymoma. PLoS ONE.

[70] Birks D.K., Barton V.N., Donson A.M., Handler M.H., Vibhakar R., Foreman N.K. (2011). Survey of microrna expression in pediatric brain tumors. Pediatric Blood Cancer.

[71] Ruiz Esparza-Garrido R., Velazquez-Flores M.A., Diegoperez-Ramirez J., Lopez-Aguilar E., Siordia-Reyes G., Hernandez-Ortiz M., Martinez-Batallar A.G., Encarnacion-Guevara S., Salamanca-Gomez F., Arenas-Aranda D.J. (2013). A proteomic approach of pediatric astrocytomas: Mirnas and network insight. J. Proteom..

[72] Appin C.L., Brat D.J. (2014). Molecular genetics of gliomas. Cancer J..

[73] López G.Y., Van Ziffle J., Onodera C., Grenert J.P., Yeh I., Bastian B.C., Clarke J., Oberheim Bush N.A., Taylor J., Chang S. (2019). The genetic landscape of gliomas arising after therapeutic radiation. Acta Neuropathol..

[74] Paugh B.S., Zhu X., Qu C., Endersby R., Diaz A.K., Zhang J., Bax D.A., Carvalho D., Reis R.M., Onar-Thomas A. (2013). Novel oncogenic pdgfra mutations in pediatric high-grade gliomas. Cancer Res..

[75] Fan R. (2014). Pax immunoreactivity in poorly differentiated small round cell tumors of childhood. Fetal Pediatric Pathol..

[76] Su W., Hopkins S., Nesser N.K., Sopher B., Silvestroni A., Ammanuel S., Jayadev S., Möller T., Weinstein J., Garden G.A. (2014). The p53 transcription factor modulates microglia behavior through microrna-dependent regulation of c-maf. J. Immunol..

[77] Morokoff A., Jones J., Nguyen H., Ma C., Lasocki A., Gaillard F., Bennett I., Luwor R., Stylli S., Paradiso L. (2020). Serum microrna is a biomarker for post-operative monitoring in glioma. J. Neuro-Oncol..

[78] Duan J., Zhou K., Tang X., Duan J., Zhao L. (2016). MicroRNA-34a inhibits cell proliferation and induces cell apoptosis of glioma cells via targeting of bcl-2. Mol. Med. Rep..

[79] Fan Y.N., Meley D., Pizer B., Sée V. (2014). Mir-34a mimics are potential therapeutic agents for p53-mutated and chemo-resistant brain tumour cells. PLoS ONE.

[80] Gao H., Zhao H., Xiang W. (2013). Expression level of human mir-34a correlates with glioma grade and prognosis. J. Neuro-Oncol..

[81] Li Q., Wang C., Cai L., Lu J., Zhu Z., Wang C., Su Z., Lu X. (2019). Mir-34a derived from mesenchymal stem cells stimulates senescence in glioma cells by inducing DNA damage. Mol. Med. Rep..

[82] Li S.Z., Hu Y.Y., Zhao J., Zhao Y.B., Sun J.D., Yang Y.F., Ji C.C., Liu Z.B., Cao W.D., Qu Y. (2014). Microrna-34a induces apoptosis in the human glioma cell line, a172, through enhanced ros production and nox2 expression. Biochem. Biophys. Res. Commun..

[83] Mikkelsen L.H., Andersen M.K., Andreasen S., Larsen A.C., Tan Q., Toft P.B., Wadt K., Heegaard S. (2019). Global microrna profiling of metastatic conjunctival melanoma. Melanoma Res..

[84] Ofek P., Calderón M., Mehrabadi F.S., Krivitsky A., Ferber S., Tiram G., Yerushalmi N., Kredo-Russo S., Grossman R., Ram Z. (2016). Restoring the oncosuppressor activity of microrna-34a in glioblastoma using a polyglycerol-based polyplex. Nanomed. Nanotechnol. Biol. Med..

[85] Rathod S.S., Rani S.B., Khan M., Muzumdar D., Shiras A. (2014). Tumor suppressive mirna-34a suppresses cell proliferation and tumor growth of glioma stem cells by targeting akt and wnt signaling pathways. FEBS Open Bio.

[86] Sakata J., Sasayama T., Tanaka K., Nagashima H., Nakada M., Tanaka H., Hashimoto N., Kagawa N., Kinoshita M., Nakamizo S. (2019). Microrna regulating stanniocalcin-1 is a metastasis and dissemination promoting factor in glioblastoma. J. Neuro-Oncol..

[87] Thor T., Künkele A., Pajtler K.W., Wefers A.K., Stephan H., Mestdagh P., Heukamp L., Hartmann W., Vandesompele J., Sadowski N. (2015). Mir-34a deficiency accelerates medulloblastoma formation in vivo. Int. J. Cancer.

[88] Toraih E.A., Aly N.M., Abdallah H.Y., Al-Qahtani S.A., Shaalan A.A., Hussein M.H., Fawzy M.S. (2017). Microrna-target cross-talks: Key players in glioblastoma multiforme. Tumour Biol..

[89] Wang Y., Wang L. (2017). Mir-34a attenuates glioma cells progression and chemoresistance via targeting pd-l1. Biotechnol. Lett..

[90] Werner T.V., Hart M., Nickels R., Kim Y.J., Menger M.D., Bohle R.M., Keller A., Ludwig N., Meese E. (2017). Mir-34a-3p alters proliferation and apoptosis of meningioma cells in vitro and is directly targeting smad4, frat1 and bcl2. Aging.

[91] Zhao H., Xing F., Yuan J., Li Z., Zhang W. (2020). Sevoflurane inhibits migration and invasion of glioma cells via regulating mir-34a-5p/mmp-2 axis. Life Sci..

[92] Butz H., Likó I., Czirják S., Igaz P., Korbonits M., Rácz K., Patócs A. (2011). Microrna profile indicates downregulation of the tgfβ pathway in sporadic non-functioning pituitary adenomas. Pituitary.

[93] Li R., Li X., Ning S., Ye J., Han L., Kang C., Li X. (2014). Identification of a core mirna-pathway regulatory network in glioma by therapeutically targeting mir-181d, mir-21, mir-23b, β-catenin, cbp, and stat3. PLoS ONE.

[94] Chen L., Zhang K., Shi Z., Zhang A., Jia Z., Wang G., Pu P., Kang C., Han L. (2014). A lentivirus-mediated mir-23b sponge diminishes the malignant phenotype of glioma cells in vitro and in vivo. Oncol. Rep..

[95] Kunder R., Jalali R., Sridhar E., Moiyadi A., Goel N., Goel A., Gupta T., Krishnatry R., Kannan S., Kurkure P. (2013). Real-time pcr assay based on the differential expression of micrornas and protein-coding genes for molecular classification of formalin-fixed paraffin embedded medulloblastomas. Neuro-Oncology.

[96] Chen L., Han L., Zhang K., Shi Z., Zhang J., Zhang A., Wang Y., Song Y., Li Y., Jiang T. (2012). Vhl regulates the effects of mir-23b on glioma survival and invasion via suppression of hif-1α/vegf and β-catenin/tcf-4 signaling. Neuro-Oncology.

[97] Geng J., Luo H., Pu Y., Zhou Z., Wu X., Xu W., Yang Z. (2012). Methylation mediated silencing of mir-23b expression and its role in glioma stem cells. Neurosci. Lett..

[98] Jiang J., Yang J., Wang Z., Wu G., Liu F. (2013). Tfam is directly regulated by mir-23b in glioma. Oncol. Rep..

[99] Leone V., Langella C., D’Angelo D., Mussnich P., Wierinckx A., Terracciano L., Raverot G., Lachuer J., Rotondi S., Jaffrain-Rea M.L. (2014). Mir-23b and mir-130b expression is downregulated in pituitary adenomas. Mol. Cell. Endocrinol..

[100] Cheng W., Ren X., Zhang C., Han S., Wu A. (2017). Expression and prognostic value of micrornas in lower-grade glioma depends on idh1/2 status. J. Neuro-Oncol..

[101] Hsieh T.H., Liu Y.R., Chang T.Y., Liang M.L., Chen H.H., Wang H.W., Yen Y., Wong T.T. (2018). Global DNA methylation analysis reveals mir-214-3p contributes to cisplatin resistance in pediatric intracranial nongerminomatous malignant germ cell tumors. Neuro-Oncology.

[102] Wang J., Che F., Zhang J., Zhang M., Xiao S., Liu Y., Zhou L., Su Q., You C., Lu Y. (2019). Diagnostic and prognostic potential of serum cell-free microrna-214 in glioma. World Neurosurg..

[103] Wang S., Jiao B., Geng S., Ma S., Liang Z., Lu S. (2014). Combined aberrant expression of microrna-214 and ubc9 is an independent unfavorable prognostic factor for patients with gliomas. Med. Oncol..

[104] Wang Y., Wang M., Wei W., Han D., Chen X., Hu Q., Yu T., Liu N., You Y., Zhang J. (2016). Disruption of the ezh2/mirna/β-catenin signaling suppresses aerobic glycolysis in glioma. Oncotarget.

[105] Xu C., He T., Li Z., Liu H., Ding B. (2017). Regulation of hoxa11-as/mir-214-3p/ezh2 axis on the growth, migration and invasion of glioma cells. Biomed. Pharmacother.Biomed. Pharmacother..

[106] Deshpande R.P., Panigrahi M., Chandrasekhar Y.B.V.K., Babu P.P. (2018). Profiling of micrornas modulating cytomegalovirus infection in astrocytoma patients. Neurol. Sci. Off. J. Ital. Neurol. Soc. Ital. Soc. Clin. Neurophysiol..

[107] Jiang Z., Yao L., Ma H., Xu P., Li Z., Guo M., Chen J., Bao H., Qiao S., Zhao Y. (2017). Mirna-214 inhibits cellular proliferation and migration in glioma cells targeting caspase 1 involved in pyroptosis. Oncol. Res..

[108] Tang S.L., Gao Y.L., Chen X.B. (2015). Microrna-214 targets pcbp2 to suppress the proliferation and growth of glioma cells. Int. J. Clin. Exp. Pathol..

[109] Yang J.K., Liu H.J., Wang Y., Li C., Yang J.P., Yang L., Qi X.J., Zhao Y.L., Shi X.F., Li J.C. (2019). Exosomal mir-214-5p released from glioblastoma cells modulates inflammatory response of microglia after lipopolysaccharide stimulation through targeting cxcr5. CNS Neurol. Disord. Drug Targets.

[110] Zhao C., Xu Y., Zhang Y., Tan W., Xue J., Yang Z., Zhang Y., Lu Y., Hu X. (2013). Downregulation of mir-145 contributes to lung adenocarcinoma cell growth to form brain metastases. Oncol. Rep..

[111] Zhao Z., Tan X., Zhao A., Zhu L., Yin B., Yuan J., Qiang B., Peng X. (2012). Microrna-214-mediated ubc9 expression in glioma. BMB Rep..

[112] Gao S., Chen J., Wang Y., Zhong Y., Dai Q., Wang Q., Tu J. (2018). Mir-592 suppresses the development of glioma by regulating rho-associated protein kinase. Neuroreport.

[113] Herman A., Gruden K., Blejec A., Podpečan V., Motaln H., Rožman P., Hren M., Zupančič K., Veber M., Verbovšek U. (2015). Analysis of glioblastoma patients’ plasma revealed the presence of micrornas with a prognostic impact on survival and those of viral origin. PLoS ONE.

[114] Tűzesi Á., Kling T., Wenger A., Lunavat T.R., Jang S.C., Rydenhag B., Lötvall J., Pollard S.M., Danielsson A., Carén H. (2017). Pediatric brain tumor cells release exosomes with a mirna repertoire that differs from exosomes secreted by normal cells. Oncotarget.

[115] Ames H.M., Yuan M., Vizcaíno M.A., Yu W., Rodriguez F.J. (2017). Microrna profiling of low-grade glial and glioneuronal tumors shows an independent role for cluster 14q32.31 member mir-487b. Mod. Pathol..

[116] Abdelfattah N., Rajamanickam S., Panneerdoss S., Timilsina S., Yadav P., Onyeagucha B.C., Garcia M., Vadlamudi R., Chen Y., Brenner A. (2018). Mir-584-5p potentiates vincristine and radiation response by inducing spindle defects and DNA damage in medulloblastoma. Nat. Commun..

[117] Song Y., Wang G., Zhuang J., Ni J., Zhang S., Ye Y., Xia W. (2019). Microrna-584 prohibits hepatocellular carcinoma cell proliferation and invasion by directly targeting bdnf. Mol. Med. Rep..

[118] Tantawy M., Elzayat M.G., Yehia D., Taha H. (2018). Identification of microrna signature in different pediatric brain tumors. Genet. Mol. Biol..

[119] Wang X.P., Deng X.L., Li L.Y. (2014). Microrna-584 functions as a tumor suppressor and targets pttg1ip in glioma. Int. J. Clin. Exp. Pathol..

[120] Xue H., Guo X., Han X., Yan S., Zhang J., Xu S., Li T., Guo X., Zhang P., Gao X. (2016). Microrna-584-3p, a novel tumor suppressor and prognostic marker, reduces the migration and invasion of human glioma cells by targeting hypoxia-induced rock1. Oncotarget.

[121] Yan W., Li R., Liu Y., Yang P., Wang Z., Zhang C., Bao Z., Zhang W., You Y., Jiang T. (2014). Microrna expression patterns in the malignant progression of gliomas and a 5-microrna signature for prognosis. Oncotarget.

[122] Hu Q., Liu F., Yan T., Wu M., Ye M., Shi G., Lv S., Zhu X. (2019). Microrna-576-3p inhibits the migration and proangiogenic abilities of hypoxia-treated glioma cells through hypoxia-inducible factor-1α. Int. J. Mol. Med..

[123] Dong L., Li Y., Han C., Wang X., She L., Zhang H. (2014). Mirna microarray reveals specific expression in the peripheral blood of glioblastoma patients. Int. J. Oncol..

[124] Xiong D.D., Xu W.Q., He R.Q., Dang Y.W., Chen G., Luo D.Z. (2019). In silico analysis identified mirna-based therapeutic agents against glioblastoma multiforme. Oncol. Rep..

[125] Calsina B., Castro-Vega L.J., Torres-Pérez R., Inglada-Pérez L., Currás-Freixes M., Roldán-Romero J.M., Mancikova V., Letón R., Remacha L., Santos M. (2019). Integrative multi-omics analysis identifies a prognostic mirna signature and a targetable mir-21-3p/tsc2/mtor axis in metastatic pheochromocytoma/paraganglioma. Theranostics.

[126] Feng S., Yao J., Zhang Z., Zhang Y., Zhang Z., Liu J., Tan W., Sun C., Chen L., Yu X. (2018). Mir-96 inhibits emt by targeting aeg-1 in glioblastoma cancer cells. Mol. Med. Rep..

[127] Gokhale A., Kunder R., Goel A., Sarin R., Moiyadi A., Shenoy A., Mamidipally C., Noronha S., Kannan S., Shirsat N.V. (2010). Distinctive microrna signature of medulloblastomas associated with the wnt signaling pathway. J. Cancer Res. Ther..

[128] Guo P., Yu Y., Tian Z., Lin Y., Qiu Y., Yao W., Zhang L. (2018). Upregulation of mir-96 promotes radioresistance in glioblastoma cells via targeting pdcd4. Int. J. Oncol..

[129] Kim Y.W., Kim E.Y., Jeon D., Liu J.L., Kim H.S., Choi J.W., Ahn W.S. (2014). Differential microrna expression signatures and cell type-specific association with taxol resistance in ovarian cancer cells. Drug Des. Dev. Ther..

[130] Minchenko D.O., Tsymbal D.O., Riabovol O.O., Viletska Y.M., Lahanovska Y.O., Sliusar M.Y., Bezrodnyi B.H., Minchenko O.H. (2019). Hypoxic regulation of edn1, ednra, ednrb, and ece1 gene expressions in ern1 knockdown u87 glioma cells. Endocr. Regul..

[131] Sun G., Ding X., Bi N., Wang Z., Wu L., Zhou W., Zhao Z., Wang J., Zhang W., Fan J. (2019). Molecular predictors of brain metastasis-related micrornas in lung adenocarcinoma. PLoS Genet..

[132] Zhang S., Zhang H., Zhu J., Zhang X., Liu Y. (2015). Mir-522 contributes to cell proliferation of human glioblastoma cells by suppressing phlpp1 expression. Biomed. Pharmacother. Biomed. Pharmacother..

[133] Ma Z. (2018). Downregulation of setd8 by mir-382 is involved in glioma progression. Pathol. Res. Pract..

[134] Song D., Diao J., Yang Y., Chen Y. (2017). Microrna-382 inhibits cell proliferation and invasion of retinoblastoma by targeting bdnf-mediated pi3k/akt signalling pathway. Mol. Med. Rep..

[135] Baertsch M.A., Leber M.F., Bossow S., Singh M., Engeland C.E., Albert J., Grossardt C., Jäger D., von Kalle C., Ungerechts G. (2014). Microrna-mediated multi-tissue detargeting of oncolytic measles virus. Cancer Gene Ther..

[136] Ding C.Q., Deng W.S., Yin X.F., Ding X.D. (2018). Mir-122 inhibits cell proliferation and induces apoptosis by targeting runt-related transcription factors 2 in human glioma. Eur. Rev. Med. Pharmacol. Sci..

[137] Fong M.Y., Zhou W., Liu L., Alontaga A.Y., Chandra M., Ashby J., Chow A., O’Connor S.T., Li S., Chin A.R. (2015). Breast-cancer-secreted mir-122 reprograms glucose metabolism in premetastatic niche to promote metastasis. Nat. Cell Biol..

[138] Su R., Cao S., Ma J., Liu Y., Liu X., Zheng J., Chen J., Liu L., Cai H., Li Z. (2017). Knockdown of sox2ot inhibits the malignant biological behaviors of glioblastoma stem cells via up-regulating the expression of mir-194-5p and mir-122. Mol. Cancer.

[139] Sun Y., Jin J.G., Mi W.Y., Zhang S.R., Meng Q., Zhang S.T. (2018). Long noncoding rna uca1 targets mir-122 to promote proliferation, migration, and invasion of glioma cells. Oncol. Res..

[140] Tang Y., Zhao S., Wang J., Li D., Ren Q., Tang Y. (2017). Plasma mir-122 as a potential diagnostic and prognostic indicator in human glioma. Neurol. Sci. Off. J. Ital. Neurol. Soc. Ital. Soc. Clin. Neurophysiol..

[141] Yerukala Sathipati S., Huang H.L., Ho S.Y. (2016). Estimating survival time of patients with glioblastoma multiforme and characterization of the identified microrna signatures. BMC Genom..

[142] Stjernfelt K.J., Von Stedingk K., Wiebe T., Hjorth L., Olsson H., Øra I. (2017). Predominance of girls with cancer in families with multiple childhood cancer cases. BMC Cancer.

[143] Picardi E., Manzari C., Mastropasqua F., Aiello I., D’Erchia A.M., Pesole G. (2015). Profiling rna editing in human tissues: Towards the inosinome atlas. Sci. Rep..

[144] Silvestris D.A., Picardi E., Cesarini V., Fosso B., Mangraviti N., Massimi L., Martini M., Pesole G., Locatelli F., Gallo A. (2019). Dynamic inosinome profiles reveal novel patient stratification and gender-specific differences in glioblastoma. Genome Biol..

[145] Liu Y., Tang K., Yan W., Wang Y., You G., Kang C., Jiang T., Zhang W. (2013). Identifying ki-67 specific mirna-mrna interactions in malignant astrocytomas. Neurosci. Lett..

[146] Kristensen B.W., Priesterbach-Ackley L.P., Petersen J.K., Wesseling P. (2019). Molecular pathology of tumors of the central nervous system. Ann. Oncol. Off. J. Eur. Soc. Med. Oncol..

[147] Muhlisch J., Schwering A., Grotzer M., Vince G.H., Roggendorf W., Hagemann C., Sorensen N., Rickert C.H., Osada N., Jurgens H. (2006). Epigenetic repression of rassf1a but not casp8 in supratentorial pnet (spnet) and atypical teratoid/rhabdoid tumors (at/rt) of childhood. Oncogene.

[148] Fleming A.J., Hukin J., Rassekh R., Fryer C., Kim J., Stemmer-Rachamimov A., Birks D.K., Huang A., Yip S., Dunham C. (2012). Atypical teratoid rhabdoid tumors (atrts): The british columbia’s children’s hospital’s experience, 1986–2006. Brain Pathol..

[149] Ebinger M., Senf L., Wachowski O., Scheurlen W. (2004). Promoter methylation pattern of caspase-8, p16ink4a, mgmt, timp-3, and e-cadherin in medulloblastoma. Pathol. Oncol. Res..

[150] Feierabend D., Walter J., Grube S., Herbold C., Beetz C., Kalff R., Ewald C. (2014). Methylation-specific multiplex ligation-dependent probe amplification and its impact on clinical findings in medulloblastoma. J. Neuro-Oncol..

[151] Harada K., Toyooka S., Maitra A., Maruyama R., Toyooka K.O., Timmons C.F., Tomlinson G.E., Mastrangelo D., Hay R.J., Minna J.D. (2002). Aberrant promoter methylation and silencing of the rassf1a gene in pediatric tumors and cell lines. Oncogene.

[152] Inda M.M., Castresana J.S. (2007). Rassf1a promoter is highly methylated in primitive neuroectodermal tumors of the central nervous system. Neuropathol. Off. J. Jpn. Soc. Neuropathol..

[153] Lindsey J.C., Lusher M.E., Anderton J.A., Bailey S., Gilbertson R.J., Pearson A.D., Ellison D.W., Clifford S.C. (2004). Identification of tumour-specific epigenetic events in medulloblastoma development by hypermethylation profiling. Carcinogenesis.

