# Supplementary material for: Differential and Common Signatures of miRNA Expression and Methylation in Childhood Central Nervous System Malignancies: An Experimental and Computational Approach
Source: Cancers (Basel). 2021 Oct 31;13(21):5491. doi: 10.3390/cancers13215491 (PMC8583574; doi:10.3390/cancers13215491)
Supplement: Supplementary file 1 [file cancers-13-05491-s001.zip › Figure S2.pdf]

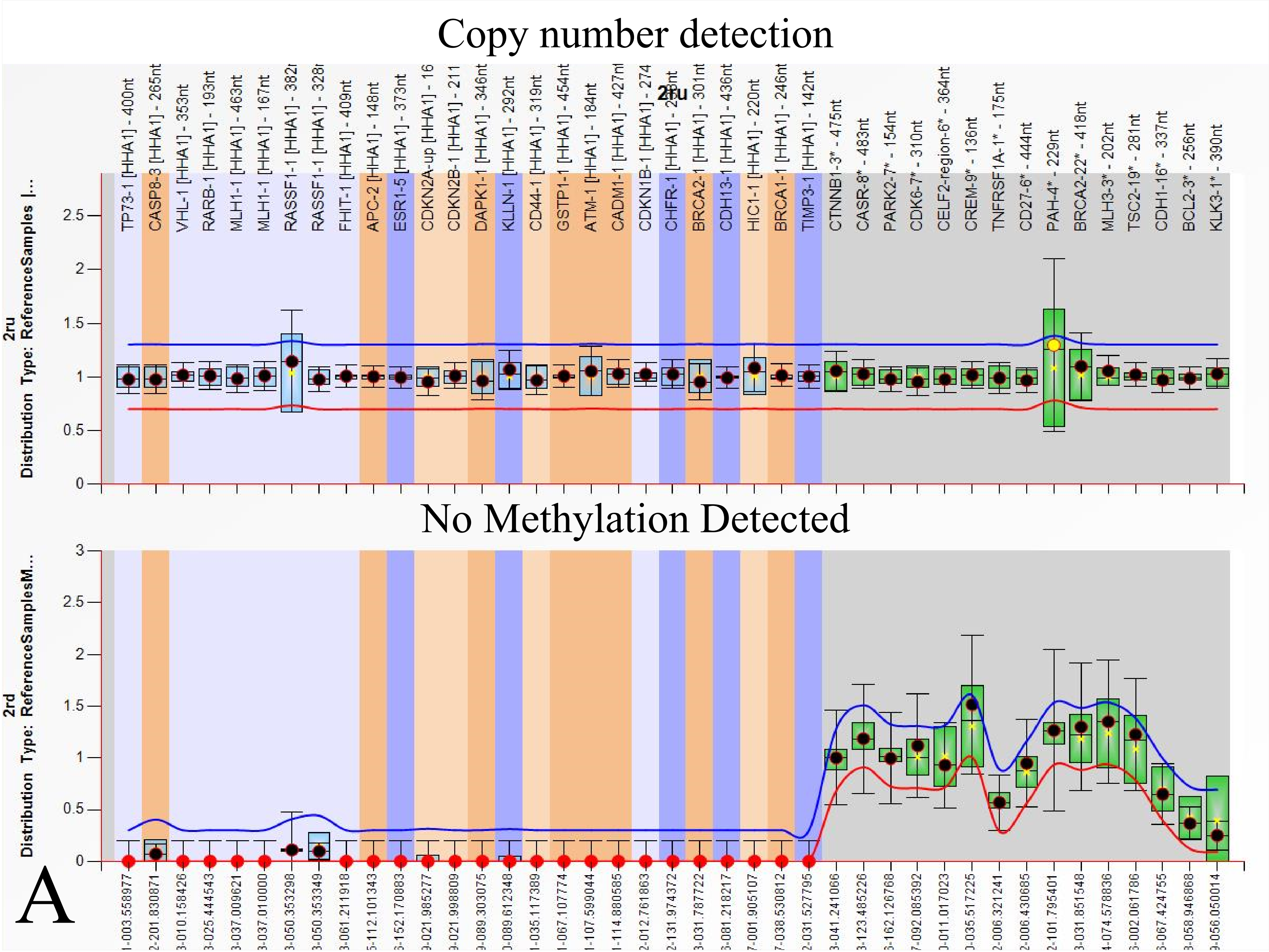
Copy number detection
2ru
Distribution Type: ReferenceSamples |...
TP73-1 [HHA1] - 400nt
CASP8-3 [HHA1] - 265nt
VHL-1 [HHA1] - 353nt
RARB-1 [HHA1] - 193nt
MLH1-1 [HHA1] - 463nt
MLH1-1 [HHA1] - 167nt
RASSF1-1 [HHA1] - 382
RASSF1-1 [HHA1] - 328
FHIT-1 [HHA1] - 409nt
APC-2 [HHA1] - 148nt
ESR1-5 [HHA1] - 373nt
CDKN2A-up [HHA1] - 16
CDKN2B-1 [HHA1] - 211
DAPK1-1 [HHA1] - 346nt
KLLN-1 [HHA1] - 292nt
CD44-1 [HHA1] - 319nt
GSTP1-1 [HHA1] - 454nt
ATM-1 [HHA1] - 184nt
CADM1-1 [HHA1] - 427nt
CDKN1B-1 [HHA1] - 274
CHFR-1 [HHA1]
BRCA2-1 [HHA1] - 301nt
CDH13-1 [HHA1] - 436nt
HIC1-1 [HHA1] - 220nt
BRCA1-1 [HHA1] - 246nt
TIMP3-1 [HHA1] - 142nt
CTNNB1-3* - 475nt
CASR-8* - 483nt
PARK2-7* - 154nt
CDK6-7* - 310nt
CELF2-region-6* - 364nt
CREM-9* - 136nt
TNFRSF1A-1* - 175nt
CD27-6* - 444nt
PAH-4* - 229nt
BRCA2-22* - 418nt
MLH3-3* - 202nt
TSC2-19* - 281nt
CDH1-16* - 337nt
BCL2-3* - 256nt
KLK3-1* - 390nt
No Methylation Detected
2rd
Distribution Type: ReferenceSamplesM...
A

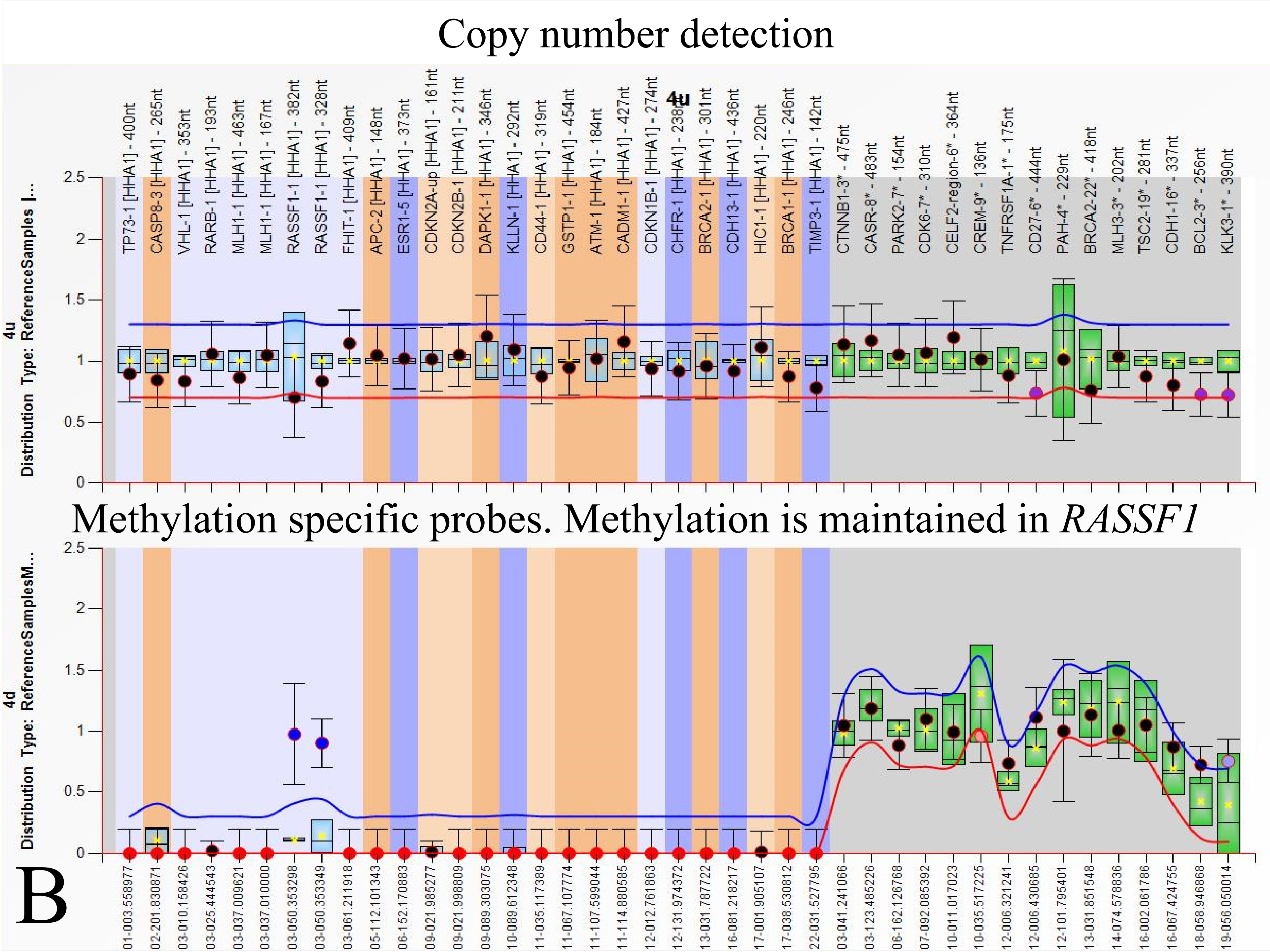
Copy number detection
4u
Distribution Type: ReferenceSamples I...
2.5
2
1.5
1
0.5
0
TP73-1 [HHA1] - 400nt
CASP8-3 [HHA1] - 265nt
VHL-1 [HHA1] - 353nt
RARB-1 [HHA1] - 193nt
MLH1-1 [HHA1] - 463nt
MLH1-1 [HHA1] - 167nt
RASSF1-1 [HHA1] - 382nt
RASSF1-1 [HHA1] - 328nt
FHIT-1 [HHA1] - 409nt
APC-2 [HHA1] - 148nt
ESR1-5 [HHA1] - 373nt
CDKN2A-up [HHA1] - 161nt
CDKN2B-1 [HHA1] - 211nt
DAPK1-1 [HHA1] - 346nt
KLLN-1 [HHA1] - 292nt
CD44-1 [HHA1] - 319nt
GSTP1-1 [HHA1] - 454nt
ATM-1 [HHA1] - 184nt
CADM1-1 [HHA1] - 427nt
CDKN1B-1 [HHA1] - 274nt
CHFR-1 [HHA1] - 238nt
BRCA2-1 [HHA1] - 301nt
CDH13-1 [HHA1] - 436nt
HIC1-1 [HHA1] - 220nt
BRCA1-1 [HHA1] - 246nt
TIMP3-1 [HHA1] - 142nt
CTNNB1-3* - 475nt
CASR-8* - 483nt
PARK2-7* - 154nt
CDK6-7* - 310nt
CELF2-region-6* - 364nt
CREM-9* - 136nt
TNFRSF1A-1* - 175nt
CD27-6* - 444nt
PAH-4* - 229nt
BRCA2-22* - 418nt
MLH3-3* - 202nt
TSC2-19* - 281nt
CDH1-16* - 337nt
BCL2-3* - 256nt
KLK3-1* - 390nt
Methylation specific probes. Methylation is maintained in *RASSF1*
4d
Distribution Type: ReferenceSamplesM...
01-003.558977
02-201.830871
03-010.158426
03-025.444543
03-037.009621
03-037.010000
03-050.353298
03-050.353349
03-061.211918
05-112.101343
06-152.170883
09-021.985277
09-021.998809
09-089.303075
10-089.612348
11-035.117389
11-067.107774
11-107.599044
11-114.880585
12-012.761863
12-131.974372
13-031.787722
16-081.218217
17-001.905107
17-038.530812
22-031.527795
03-041.241066
03-123.485226
06-162.126768
07-092.085392
10-011.017023
10-035.517225
12-006.321241
12-006.430685
12-101.795401
13-031.851548
14-074.578836
16-002.061786
16-067.424755
18-058.946868
19-056.050014
B

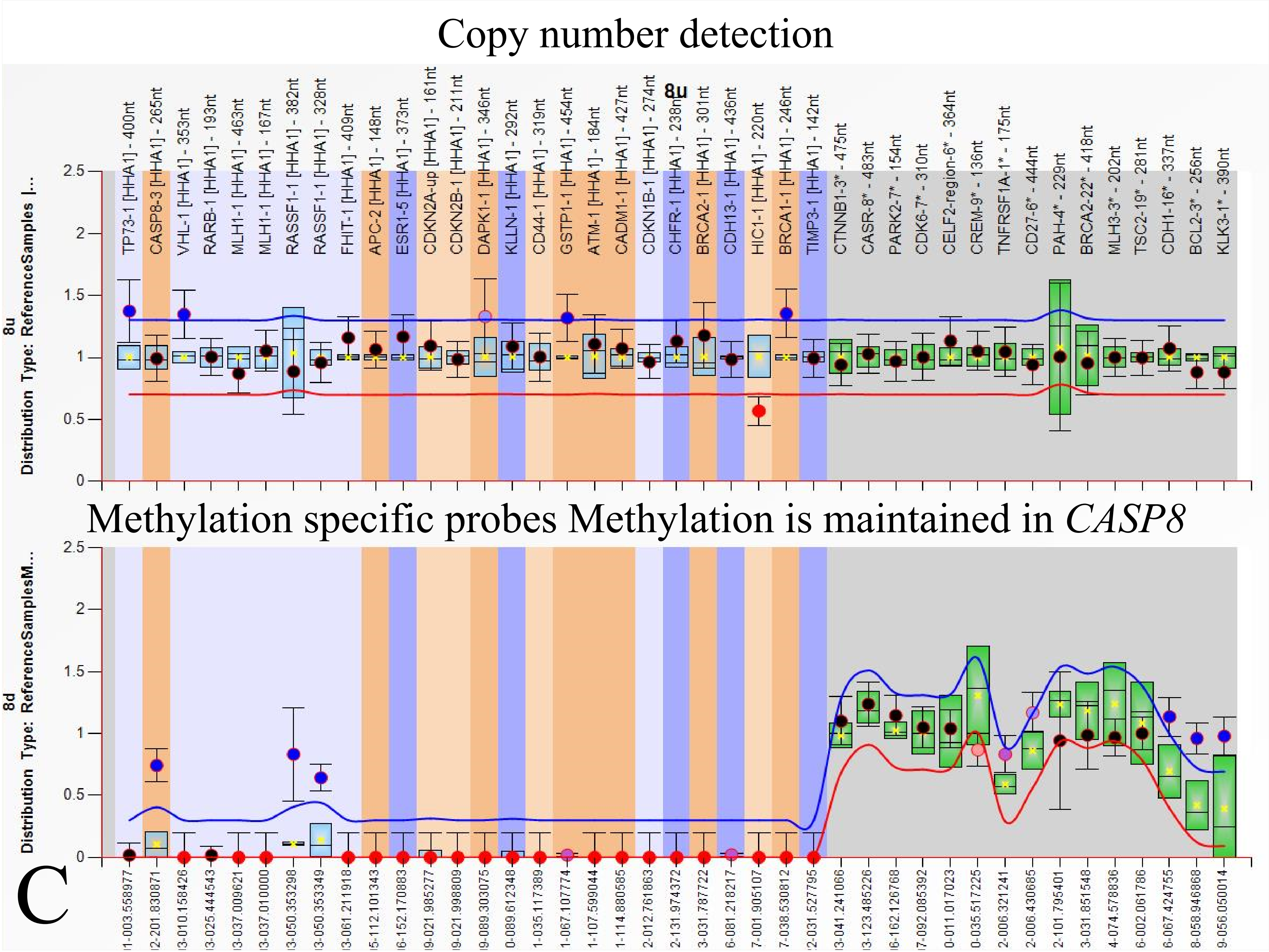
Copy number detection
8u
Distribution Type: ReferenceSamples |...
TP73-1 [HHA1] - 400nt
CASP8-3 [HHA1] - 265nt
VHL-1 [HHA1] - 353nt
RARB-1 [HHA1] - 193nt
MLH1-1 [HHA1] - 463nt
MLH1-1 [HHA1] - 167nt
RASSF1-1 [HHA1] - 382nt
RASSF1-1 [HHA1] - 328nt
FHIT-1 [HHA1] - 409nt
APC-2 [HHA1] - 148nt
ESR1-5 [HHA1] - 373nt
CDKN2A-up [HHA1] - 161nt
CDKN2B-1 [HHA1] - 211nt
DAPK1-1 [HHA1] - 346nt
KLLN-1 [HHA1] - 292nt
CD44-1 [HHA1] - 319nt
GSTP1-1 [HHA1] - 454nt
ATM-1 [HHA1] - 184nt
CADM1-1 [HHA1] - 427nt
CDKN1B-1 [HHA1] - 274nt
CHFR-1 [HHA1] - 238nt
BRCA2-1 [HHA1] - 301nt
CDH13-1 [HHA1] - 436nt
HIC1-1 [HHA1] - 220nt
BRCA1-1 [HHA1] - 246nt
TIMP3-1 [HHA1] - 142nt
CTNNB1-3* - 475nt
CASR-8* - 483nt
PARK2-7* - 154nt
CDK6-7* - 310nt
CELF2-region-6* - 364nt
CREM-9* - 136nt
TNFRSF1A-1* - 175nt
CD27-6* - 444nt
PAH-4* - 229nt
BRCA2-22* - 418nt
MLH3-3* - 202nt
TSC2-19* - 281nt
CDH1-16* - 337nt
BCL2-3* - 256nt
KLK3-1* - 390nt
Methylation specific probes Methylation is maintained in CASP8
8d
Distribution Type: ReferenceSamplesM...
C

Copy number detection

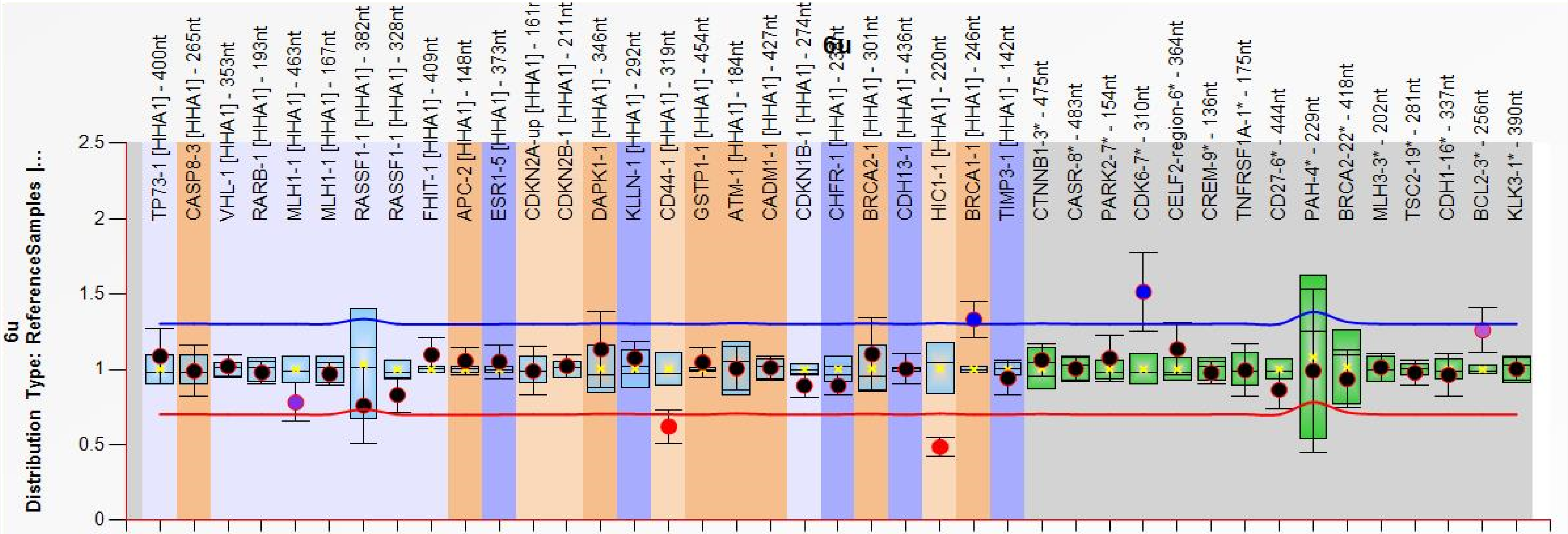

Methylation specific probes Methylation is maintained in *CASP8* and *RASSF1*

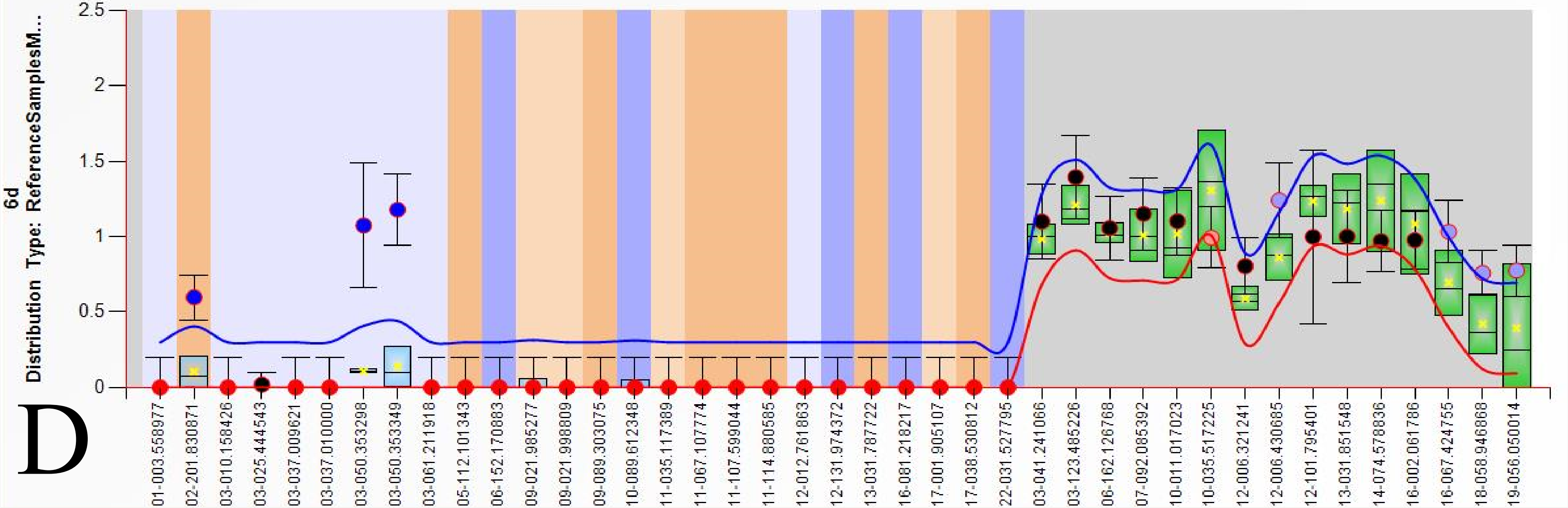

D
