# Supplementary figures and images for: Differential and Common Signatures of miRNA Expression and Methylation in Childhood Central Nervous System Malignancies: An Experimental and Computational Approach
Source: Cancers (Basel). 2021 Oct 31;13(21):5491. doi: 10.3390/cancers13215491 (PMC8583574; doi:10.3390/cancers13215491)

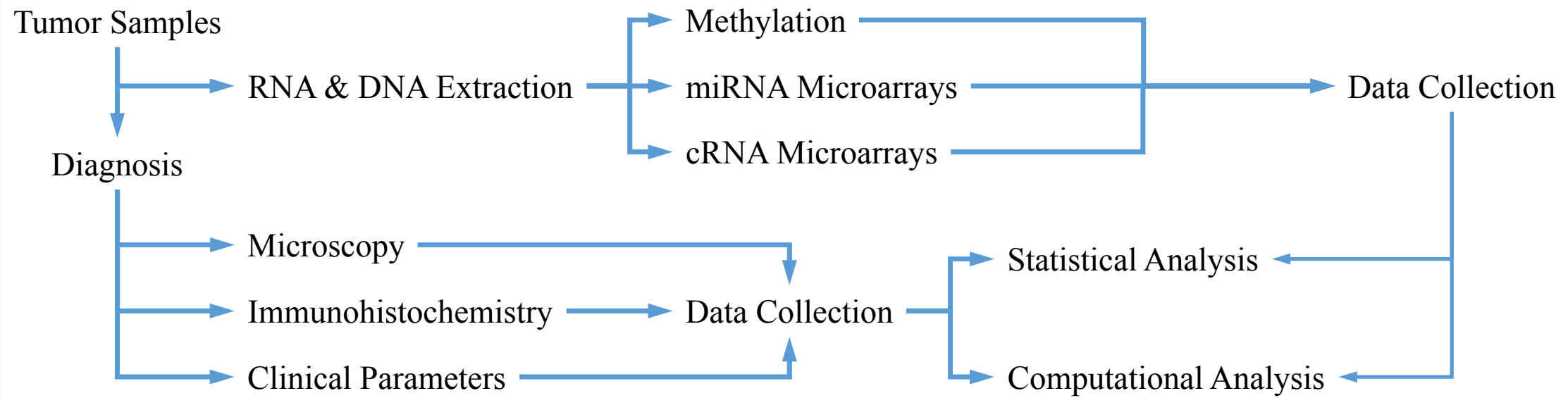

Supplement: Supplementary file 1 [file cancers-13-05491-s001.zip › Figure S1.pdf]
